# Interaction between DNA, Albumin and Apo-Transferrin and Iridium(III) Complexes with Phosphines Derived from Fluoroquinolones as a Potent Anticancer Drug

**DOI:** 10.3390/ph14070685

**Published:** 2021-07-16

**Authors:** Sandra Amanda Kozieł, Monika Katarzyna Lesiów, Daria Wojtala, Edyta Dyguda-Kazimierowicz, Dariusz Bieńko, Urszula Katarzyna Komarnicka

**Affiliations:** 1 Faculty of Chemistry, University of Wroclaw, Joliot-Curie 14, 50-383 Wroclaw, Poland; monika.lesiow@chem.uni.wroc.pl (M.K.L.); daria.wojtala@wp.pl (D.W.); 2 Faculty of Chemistry, Wroclaw University of Science and Technology, Wybrzeże Wyspiańskiego 27, 50-370 Wroclaw, Poland; Edyta.Dyguda@pwr.edu.pl (E.D.-K.); dariusz.bienko@pwr.edu.pl (D.B.)

**Keywords:** arene iridium(III) complexes, fluoroquinolones, DNA-binding studies, protein-binding studies, drug delivery, reactive oxygen species

## Abstract

A group of cytotoxic half-sandwich iridium(III) complexes with aminomethyl(diphenyl)phosphine derived from fluoroquinolone antibiotics exhibit the ability to (i) accumulate in the nucleus, (ii) induce apoptosis, (iii) activate caspase-3/7 activity, (iv) induce the changes in cell cycle leading to G2/M phase arrest, and (v) radicals generation. Herein, to elucidate the cytotoxic effects, we investigated the interaction of these complexes with DNA and serum proteins by gel electrophoresis, fluorescence spectroscopy, circular dichroism, and molecular docking studies. DNA binding experiments established that the complexes interact with DNA by moderate intercalation and predominance of minor groove binding without the capability to cause a double-strand cleavage. The molecular docking study confirmed two binding modes: minor groove binding and threading intercalation with the fluoroquinolone part of the molecule involved in pi stacking interactions and the Ir(III)-containing region positioned within the major or minor groove. Fluorescence spectroscopic data (HSA and apo-Tf titration), together with molecular docking, provided evidence that Ir(III) complexes can bind to the proteins in order to be transferred. All the compounds considered herein were found to bind to the tryptophan residues of HSA within site I (subdomain II A). Furthermore, Ir(III) complexes were found to dock within the apo-Tf binding site, including nearby tyrosine residues.

## 1. Introduction

There is a massive development in exploiting the medicinal properties of organometallic compounds in recent years, which have become an alternative to the clinically used anticancer drugs based on platinum (e.g., cisplatin, carboplatin, nedaplatin). These organometallic complexes with novel mechanisms of action have the ability to broaden the spectrum approach to tumors, reduce side effects, and overcome drug resistance [1,2,3]. The iridium(III) organometallic complexes have been rising stars and possess unique properties such as potential redox features, universal structure, wide range of ligand substitution rates, higher cellular uptake efficiency, large stokes shifts and lower toxicity [4,5,6,7,8]. Moreover, because of the fundamental differences between the chemistry of iridium and platinum complexes, one can also expect diverse mechanisms of action [9]. Furthermore, combining two or even more multifunctional structural elements brings into play different properties of a compound and may result in improving the spectrum of biological activity, novel mechanisms of action, or modification of the pharmacokinetic profile of the drug [4,5,6,7,8,9]. For example, half-sandwich Ir(III) compounds containing phosphines derived from fluoroquinolones can be outstanding examples of this popular strategy of combining structural elements currently being used to design new therapeutics. It is worth noting that quinolones are currently undergoing many structural modifications, including the formation of coordination compounds, aimed not only at overcoming the growing antibiotic resistance of microbes but also potential alternatives to established anticancer chemotherapeutic agents [10]. This paper is a continuation of our ongoing projects focused on cytotoxic activity of iridium(III) complexes bearing phosphines derived from fluoroquinolones: ciprofloxacin (HCp) [11], sparfloxacin (HSf) [12], lomefloxacin (HLm) [13], and norfloxacin (HNr) [11].

Since deoxyribonucleic acid plays a significant role in storing and expressing genetic information in a cell [13,14,15], it is the main target for several anticancer medicines, steroids, and several classes of drug [3]. Many small molecules exert their anticancer activities via binding with DNA, which usually causes damage to DNA in cancerous cells, inhibiting their division, and finally leading to cell death [1,3]. Moreover, most antibiotics and anticancer drugs tend to interact with DNA noncovalently through three selective modes: (i) A groove-bound fashion (minor or major grooves) stabilized by a mixture of hydrophobic, electrostatic, and hydrogen-bonding interactions; (ii) an intercalative association of planar, heteroaromatic moieties between the DNA base pairs and (iii) electrostatic binding [16]. Therefore, DNA-binding studies of Ir(III) complexes performed by us may be valuable in recognizing a specific site of interaction or conformation of deoxyribonucleic acid, which would help to understand the precise mode of their cytotoxic action.

Furthermore, there have been numerous reports on the investigation of interactions between serum proteins with metal complexes such as Cu^II^ [17,18], Ru^III^, Ru^II^ [19,20], Pt^IV^, or Pt^II^ [21,22]. In general, linking proteins with metal complexes can change their biological activity. For example, some in vitro and in vivo studies have shown that treatment of cancer cells with albumin-NAMI-A or transferrin-NAMI-A adducts (NAMI-A: (ImH)[trans-RuCl(4)(dmso)(Im)], Im is imidazole), resulting in a significant reduction in biological activity compared with the parent ruthenium complex [23]. On the other hand, the transfer of another ruthenium compound, KP1019 (trans-[tetrachlorobis(1*H*-indazole)ruthenate(III)]), to the cell was promoted by transferrin. Furthermore, cell fractionation studies showed that after only 2 h of exposure as much as 55% of the intracellular KP1019 was found in the nuclear fraction [24]. In this case, the biological activity increased significantly. Thus, investigating the binding of an anticancer drug with biomacromolecules is the first step to understanding the corresponding mechanism. In addition, the apparent volume of distribution and the rate of drug elimination are influenced by drug interactions at the protein binding level [25]. Biomacromolecules perform several significant functions necessary in normal biological processes that ensure their proper functioning, establishing them as an important field of research in chemistry and medicine.

Human serum albumin (HSA) is one of the most abundant proteins in plasma, accounting for 55–65% of all blood proteins. HSA is involved in the transport of metal ions and metal complexes with drugs through the bloodstream [13,26,27,28]. Additionally, albumin has shown remarkable promise as a carrier for anticancer agents based on several key characteristics: (1) The ability to extend the half-life of rapidly cleared medicines in the bloodstream; (2) the capability of interacting with different molecules that could be either peptidyl or non-peptidyl in structure; (3) increased uptake and metabolism by fast-growing, nutrient-starved cancer cells; (4) the potential to increase the uptake of the medicine by cancer cells; or, (5) on the other hand, slow down or prevent it from reaching the target tissues. Albumin is a remarkable carrier protein that has the potential to overcome barriers to the delivery of many promising anticancer agents. The report on plasma protein binding is currently accepted as an FDA (The United States Food and Drug Administration) requirement for the early screening of a potential therapeutic agent [21,29,30].

Transferrin (Tf) is responsible for the mobilization of iron by binding two Fe^3+^ ions in sites containing two tyrosine residues, a single histidine and asparagine residues, and a carbonate ion, in an overall octahedral environment [31,32]. Transferrin (Tf) transports iron ions to virtually all tissues through the transferrin receptor (TfR, CD71), which is located on cell surfaces. Expression of the TfR is significantly upregulated in a variety of cancer cells, and in many cases, increased expression correlates with tumor stage and is associated with poor prognosis. It is expressed more abundantly in cancer tissues than in normal ones because of the higher iron demand for faster cell growth and division of the cancer cells [33,34,35]. Based on this fact, transferrin can be an attractive natural carrier for anticancer metal ions [21,36,37] and other chemotherapeutic drugs [38,39]. In blood serum, only 30% of transferrin remains in holo-form while 70% stays in its apo-form (apo-Tf) and can be utilized for binding various ions at the same specific sites as iron [16,34].

The information presented above encouraged our group to explore the interactions of DNA-iridium(III) complexes and protein-iridium(III) complexes in more detail. To realize our goal, we undertook a series of experiments: (i) We tested the ability of iridium(III) complexes to interact with DNA (intercalation, major or minor groove binding) using different methods, e.g., fluorescence spectroscopy, gel electrophoresis, and molecular docking; (ii) to monitor structural changes of DNA, circular dichroism (CD) study was performed; additionally, (iii) to evaluate the ability of studied complexes to HSA and apo-Tf binding, we applied different approaches based on fluorescence spectroscopy, circular dichroism (CD) and molecular docking.

## 2. Results and Discussion

### 2.1. Synthesis, Physicochemical and Biological Characterization

**Scheme 1:**
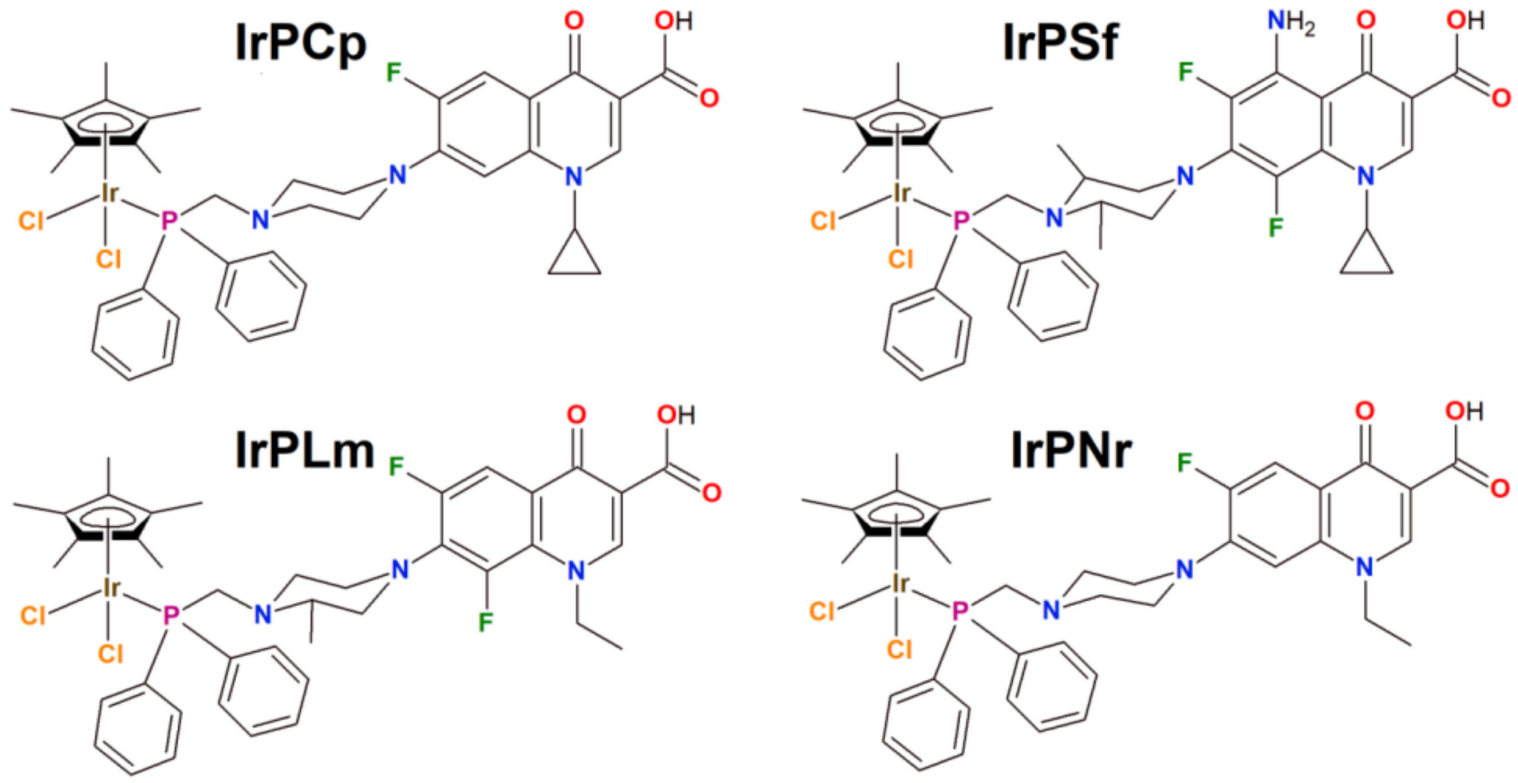
Organometallic iridium(III) complexes used in this study.

In this paper, we used four half-sandwich Ir(III) complexes (**IrPCp—**Ir(η^5^-Cp*)Cl_2_**PCp**, **IrPSf—**Ir(η^5^-Cp*)Cl_2_**PSf**, **IrPLm—**Ir(η^5^-Cp*)Cl_2_**PLm**, **IrPNr—**Ir(η^5^-Cp*)Cl_2_**PNr,** where Cp* is pentamethylcyclopentadienyl) with phosphines conjugated with fluoroquinolones as ligands, which were synthesized according to the literature procedure described by our group in previous publications (Scheme 1) [11,12,13,14]. All complexes were precisely characterized by selected methods, i.e., absorption and fluorescence spectroscopies, ESI-MS, NMR, and electrochemical techniques.

**Figure 1:**
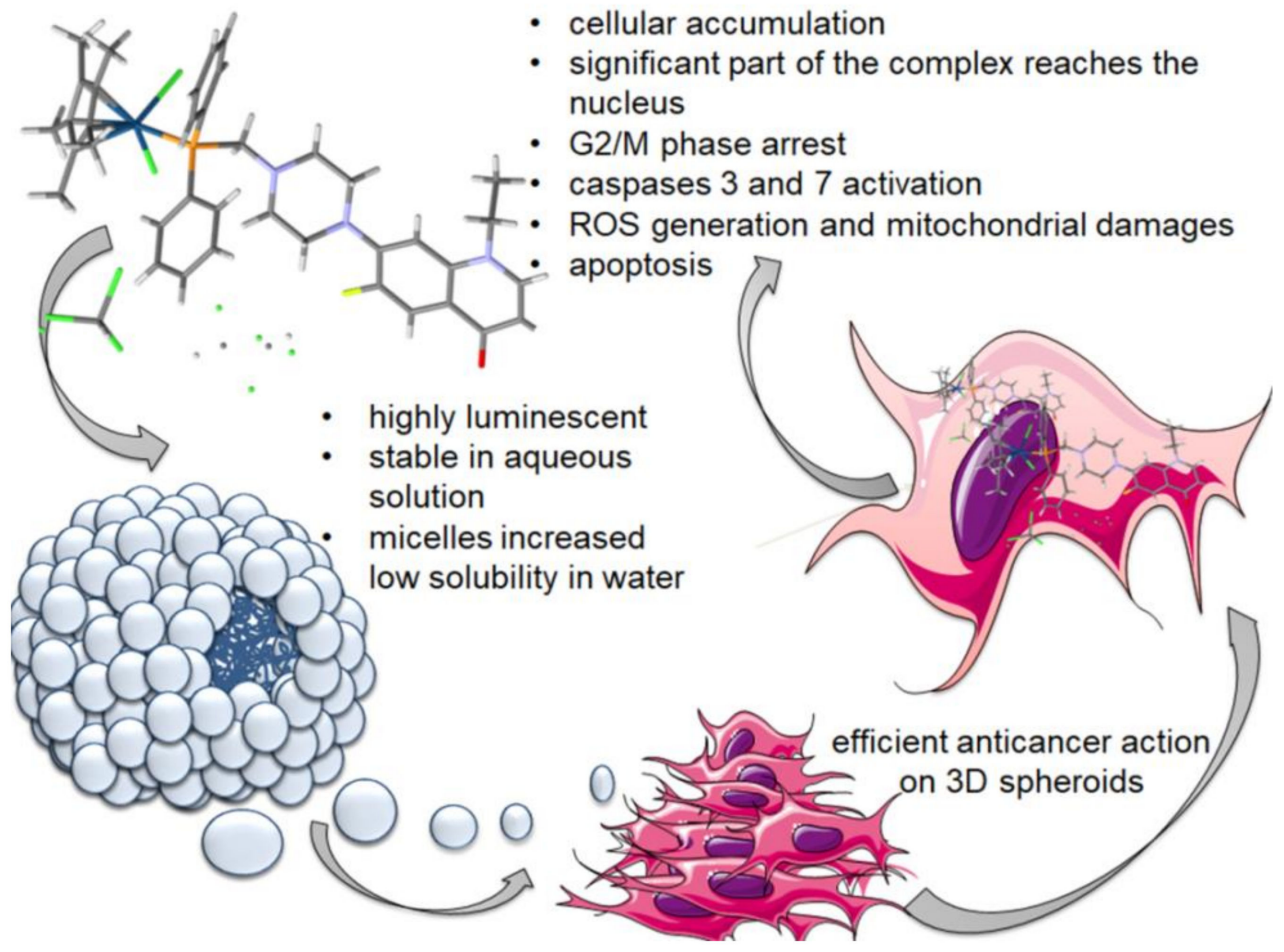
Scheme of cytotoxic action mechanism of Ir(III) complexes described by our group in a previous paper (Kozieł, S. et al., Inorganic Chemistry Frontiers, 2020, 7, 3386–3401).

The products of all syntheses were recrystallized in order to obtain pure complexes. Their purity was confirmed using elemental analysis, while the single crystals were colored by the X-ray diffraction technique (Figure 1) [14]. Additionally, long-term stability monitoring was performed using UV-Vis spectroscopy in DMEM (Dulbecco’s Modified Eagle’s Medium) with 2% DMSO. During 72 h of incubation, no significant changes in the intensity and shape of the characteristic absorption band (MLCT) were observed.

It is noteworthy that the analysis of the luminescence spectra showed that **IrPCp**, **IrPNr** and **IrPLm** are characterized by a red luminescence. Only in the case of **IrPSf,** the blue luminescence was noticed. It should be noted that the complexes’ fluorescent property provides useful information about the distribution, accumulation, and uptake of the anticancer drugs in living cells or organisms. In addition, we studied their cytotoxic effects in vitro toward selected cancer cell lines: CT26 (mouse colon carcinoma), A549 (human lung adenocarcinoma), MCF7 (human breast adenocarcinoma), PANC-1 (human pancreatic/duct carcinoma), DU-145 (human prostate carcinoma), and normal HEK293T (human embryonic kidney) cell line (Figure 1) [14].

We demonstrated in vitro that the studied complexes are characterized by lower IC_50_ values than cisplatin, exhibit the ability to induce apoptotic cell death in predominance, and possess a high therapeutic index. Additionally, preliminary investigation on the mode of action in the selected Ir(III) compounds allowed us to formulate the following conclusions: (i) Pearson’s co-localization coefficient of 0.63 indicates a uniform distribution of complexes in both the nucleus and cytoplasm; (ii) the tested compounds presumably induce G2/M phase arrest; (iii) both the activation of caspase-3/7 and the decrease of mitochondrial membrane potential confirm the apoptotic pathway of cell death; and (iv) redox potentials enable efficient ROS generation [14]. Many aspects of cancer-inhibiting action displayed by iridium complexes are still unknown. For this reason, in previous [14] and current papers we are trying to understand their mode of action.

### 2.2. Interaction with DNA

#### 2.2.1. Competitive Fluorescence Studies and DNA Degeneration

**Figure 2:**
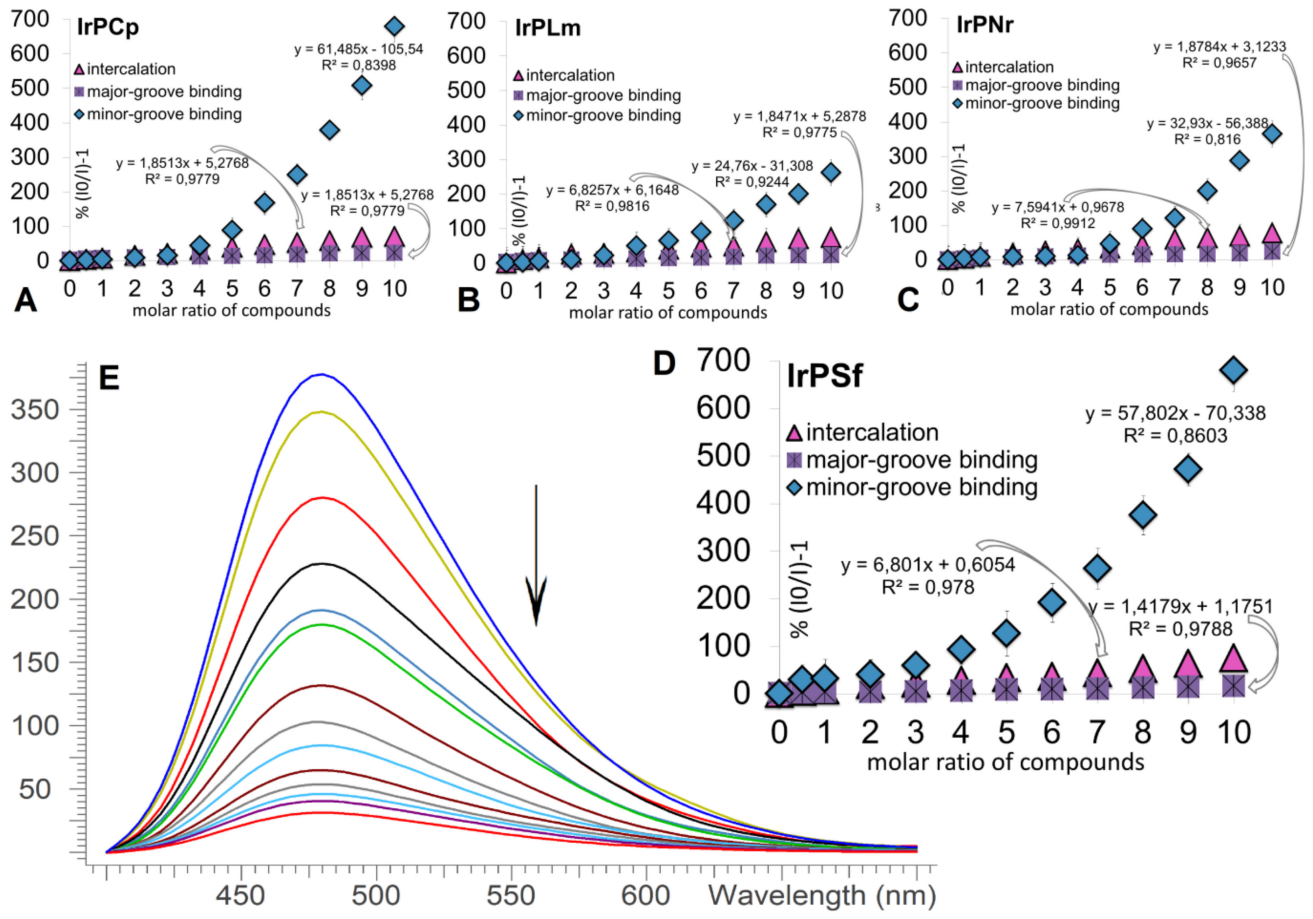
Stern–Volmer plots of the CT DNA-EB (intercalation), CT DNA-DAPI (binding to a minor groove) and CT DNA-MG (binding to a major groove), system quenched by (**A**) **IrPCp**; (**B**) **IrPLm**; (**C**) **IrPNr**; (**D**) **IrPSf**; (I_0_ and I—intensity of CT DNA-EB or DAPI or MG in the absence and the presence of increasing concentration [mM] of the compounds; (**E**) Fluorescence quenching of DAPI-DNA (C = 5 × 10^−5^ M) by **IrPSf** (molar ratios 0.5–10) in 50 mM pH 7.4 phosphate buffer (axis: y—fluorescence intensity; x—wavelength).

It is well known that the cytotoxic activity of various drugs (e.g., fluoroquinolones) depends on interaction with DNA [1]. Since we proved that investigated Ir(III) complexes can accumulate inside of the cancer nucleus, we decided to study the mode of Ir(III) complex-DNA interactions. Several well-known DNA binding dyes with well-established binding modes were used: ethidium bromide (EB; intercalation), 4′,6-diamidino-2-phenylindole (DAPI; binding to a minor groove), and methyl green (MG; binding to a major groove). The compounds that can bind to DNA more strongly than the above-mentioned binding dyes (EB, DAPI, or MG) reduce the DNA-binding dye emission due to the replacement of dye [40,41]. The emission spectra of the CT DNA-binding dye complex were measured at different concentrations of the investigated compounds, (Figure 2; Supplementary Materials, Figures S1–S4).

The intensity of the CT DNA-EB, CT DNA-DAPI or CT DNA-MG characteristic emission band significantly decreased with increasing molar ratio (0.5–10) for all discussed compounds (**IrPCp**, **IrPNr**, **IrPLm**, **IrPSf**, **PCp**, **PNr**, **PLm**, **PSf**) (Supplementary Materials, Figures S1–S4). This observed fluorescence quenching indicates that studied compounds intercalated between DNA base pairs and are bound in a minor groove of DNA. Due to the negligible binding of the compounds to DNA through the major groove, the corresponding results will not be discussed in detail; however, spectra and Ksv values are presented in Supplementary Figure S4 and Table 1, respectively. Observed spectral changes clearly revealed that complexes exhibited multimodal DNA interaction with the predominance of minor groove binding. In order to confirm the quenching mechanism, the fluorescence quenching was analyzed according to Stern–Volmer equations; determined Ksv values are presented in Table 1.

Worthy of note is that all noncovalent interactions of iridium(III) complexes with DNA were stronger than the interactions of the corresponding phosphine ligands. Fluorescence quenching plots for CT DNA-EB complex showed that studied Ir(III) complexes tend to interact with negatively charged DNA phosphate chains. The strength of the compounds’ interactions with DNA is similar for all complexes discussed (Figure 2; Supplementary Materials, Figure S1, Table 1). Notably, the trend in its ability to displace intercalator EB from CT DNA was different than in the case previously studied by our group regarding ruthenium(II) and copper(I) complexes based on the same phosphine ligands [11,13,41,42,43,44]. This trend suggests that the type of metal ions (Cu(I), Ru(II), Ir(III)) have a significant influence on the type and intensity of intercalations with DNA.

**Figure 3:**
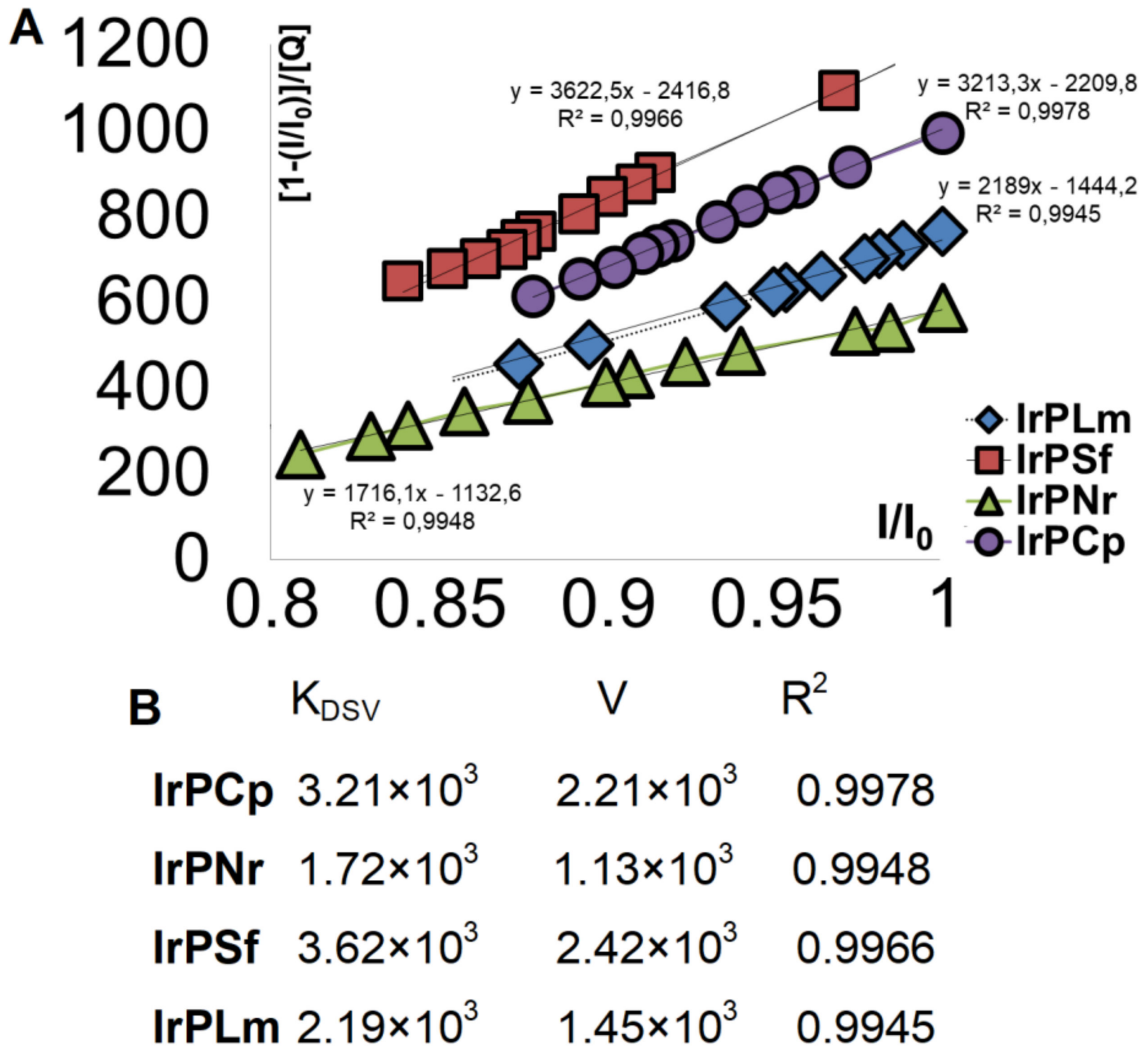
(**A**) Stern–Volmer plots of the CT DNA–DAPI (binding to a minor groove) system quenched by **IrPCp**, **IrPLm**, **IrPNr**, **IrPSf**; (**B**) determined K_DSV_ (dynamic quenching constant) values for the studied Ir(III) complexes.

Furthermore, we conducted a similar competitive experiment using DAPI Figure 3, and Figures S2 and S3 in Supplementary Materials. Observed fluorescence quenching of CT DNA-DAPI complex clearly indicates that iridium(III) complexes and their ligands are able to bind in the DNA minor groove. The displacement of bound DAPI from its binding site on CT DNA is much stronger than when EB is used. In order to confirm the quenching mechanism, the fluorescence quenching was analyzed according to Stern–Volmer equations (Figure 3A) and K_DSV_ (dynamic quenching constant) values, which are presented in Figure 3B. Based on Stern–Volmer plots (Figure 3A), the strength of CT DNA-DAPI fluorescence quenching formed the following order: **IrPSf** > **IrPCp** > **IrPNr** > **IrPLm**. This result suggests that from the perspective of more efficient binding in minor groove DNA, the presence of the cyclopropane substituent in the antibiotic structure plays a crucial role (Figure 3B).

Considering the results presented above, we can expect that the resulting complexes will not exhibit high genotoxicity, as opposed to previously reports of similarly mixed copper(I) or ruthenium(II) complexes bearing phosphines derived from fluoroquinolones [11,41,42,43,44] that exert high systemic toxicity related to strong intercalations with DNA.

**Figure 4:**
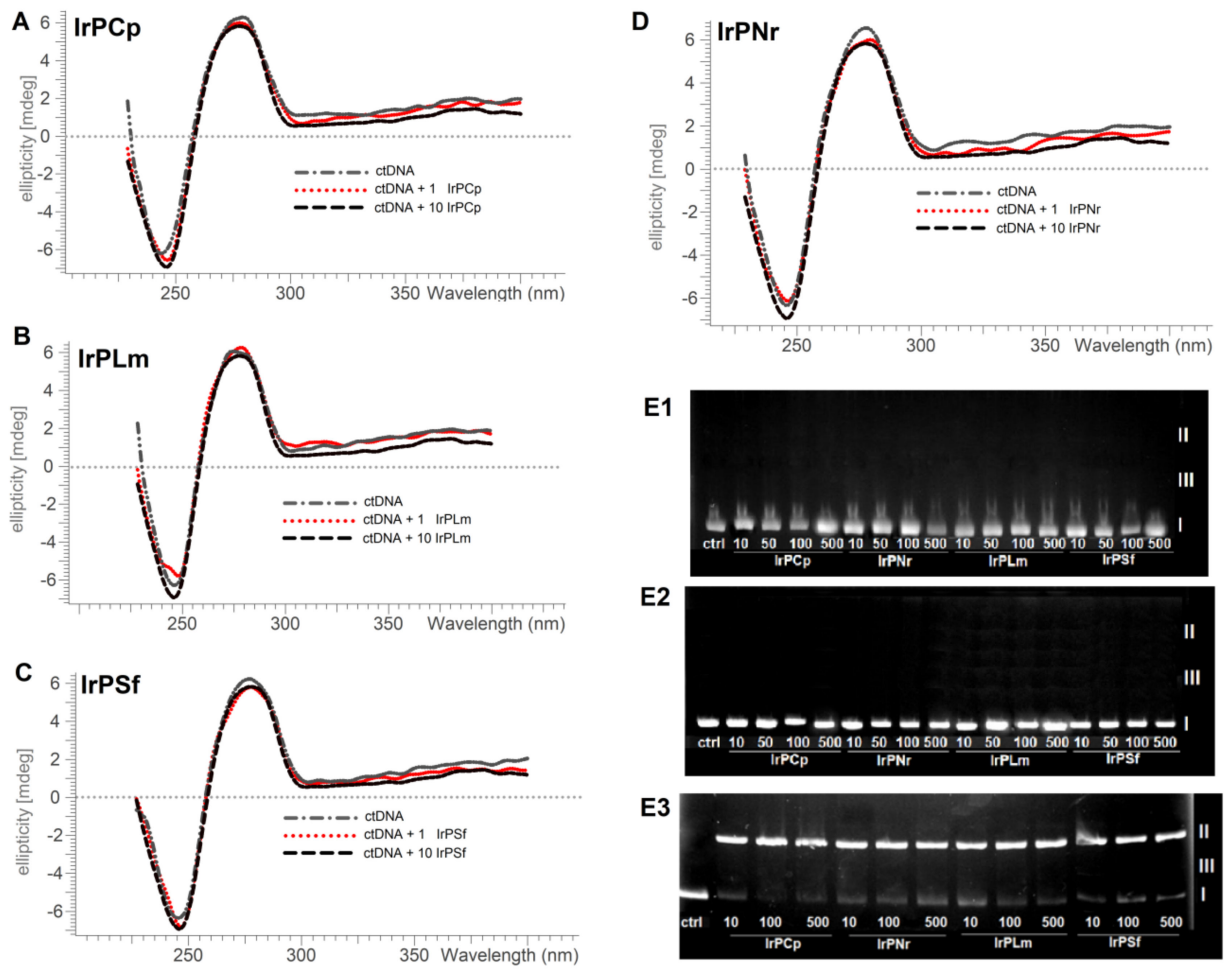
Circular dichroism spectra of CT DNA with: (**A**) **IrPCp**; (**B**) **IrPLm**; (**C**) **IrPSf**; (**D**) **IrPNr**; (**E**) agarose gel electrophoresis of pBR322 plasmid cleavage by **IrPCp**, **IrPSf**, **IrPLm** and **IrPNr** in a DMF (each in the 10% DMF) solution, ctrl: plasmid—control. (**E1**) 1 h of incubation; (**E2**) 4 h of incubation; (**E3**) 24 h of incubation.

Circular dichroism (CD) study was conducted to monitor the structural changes after the interaction of studied iridium(III) complexes with CT DNA. The CD spectrum of CT DNA exhibited two bands: a positive band at 276 nm (π–π base stacking) and a negative band appearing at 248 nm (helicity of B-DNA), Figure 4A–D [45]. The interaction between CT-DNA and complex molecules can be determined by the spectral variation of these two peaks. Upon groove binding or electrostatic binding modes, there will only be a slight spectral change in these two peaks since complex molecules do not exert a strong effect on the aggregation of DNA base pairs and DNA spiral. On the other hand, if complex molecules are inserted into DNA double helix chains, these two peaks will show noticeable spectral change [46,47]. After CT DNA titration by Ir(III) compounds, the bands slightly changed, which is consistent with the binding method suggested above—the predominance of minor groove binding. This slight change means that iridium(III) complexes did not affect DNA structure. Furthermore, these results are fully consistent with those obtained using gel electrophoresis techniques (Figure 4E1–E3).

We proved that our complexes exhibited multimodal DNA interaction (minor groove binding and intercalation). Since intercalation usually results in relatively large changes in the double helix structure, we assumed that all iridium(III) complexes cause severe DNA cleavage. The binding ability of iridium(III) complexes with another DNA model—pBR322 plasmid DNA, was studied by gel electrophoresis as a target. The degree of plasmid degradation, which is naturally present in a superhelical form (form I), to determine the ability of the compounds to induce single- and/or double-strand damage of the DNA was checked. This process can lead to the formation of the relaxed/nicked (form II) and linear (form III) forms. The degree of DNA degradation was determined in a wide range of the tested compounds’ concentrations (from 10 to 500 μM) at three different incubation times: 1 h, 4 h, and 24 h (Figure 4E1–E3).

In reality, iridium(III) complexes, the same as ligands, [11,12,13] did not cause damage to the double strand of DNA in a short incubation time (Figure 4E1,E2). It is worth noting that after only 24 h of incubation (Figure 4E3), these complexes were able to cause a single-strand plasmid cleavage. Our observation confirmed our previous results vide supra that these complexes can probably interact with DNA not only through intercalation but also different ways, e.g., covalent or different noncovalent interactions (electrostatic interactions and groove binding).

It is well-known that hydrogen peroxide is a source of hydroxyl radicals and a strong DNA oxidant in the presence of transition metal ions and their complexes, as we demonstrated in our previous studies focused on copper(I) complexes [48,49].

It was proven by cyclic voltamperometry, and fluorescence spectroscopy that the studied herein Ir(III) compounds can generate high levels of ROS, therefore the effect of additional H_2_O_2_ on the overall ROS production and the associated DNA damages caused by this radical pathway [14]. Furthermore, to confirm the generation of particular types of ROS involved in plasmid degradation, the experiment using DMSO (effective scavenger of ^•^OH), SOD (effective scavenger of O_2_^•−^), [50] and NaN_3_ (effective scavenger of ^1^O_2_) was performed [51]. In order to exclude the effect of the hydrogen peroxide influence on plasmid DNA degradation, we performed an additional gel electrophoresis for H_2_O_2_ in different concentrations (Supplementary Materials, Figure S8).

**Figure 5:**
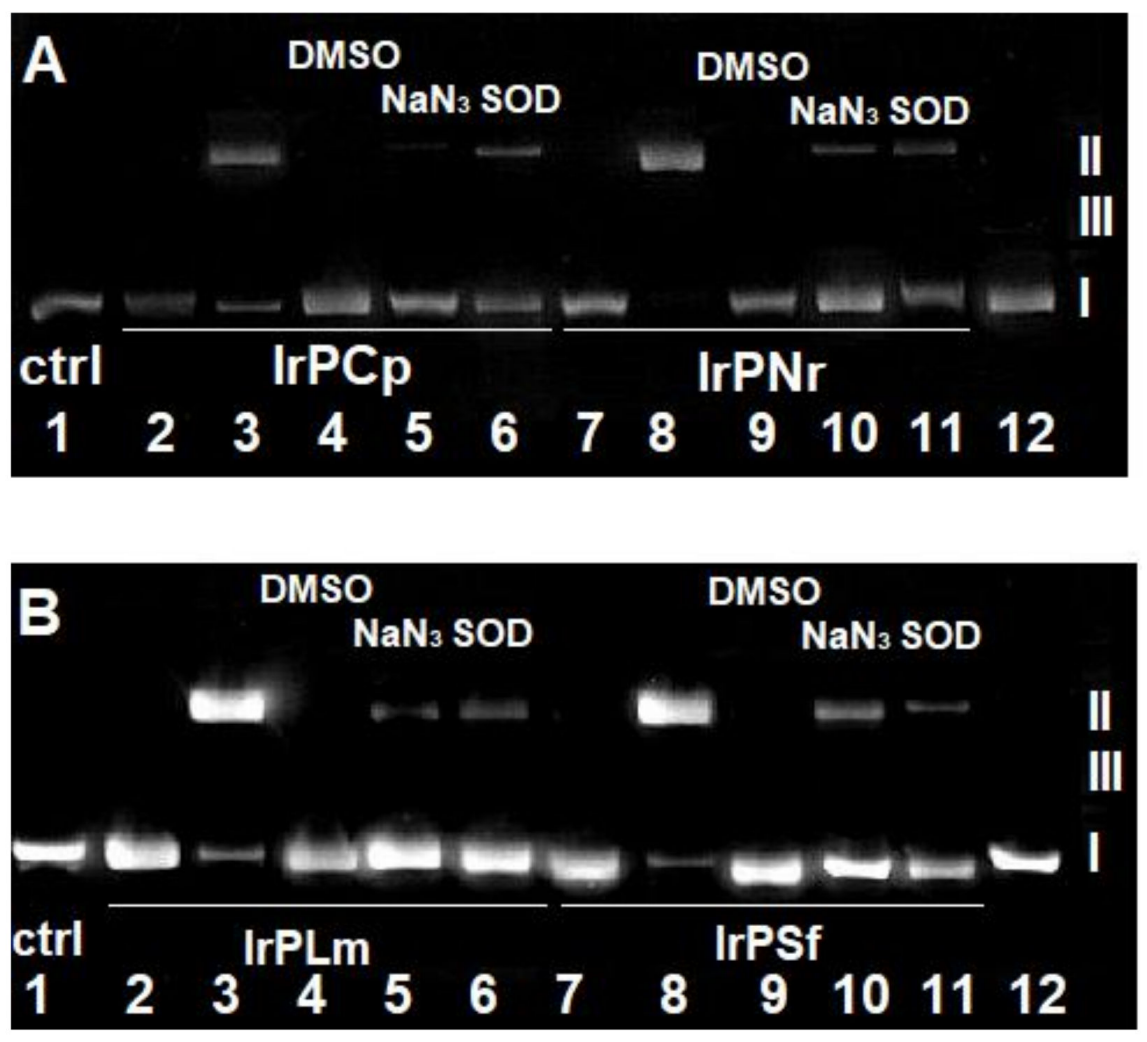
Agarose gel electrophoresis of pBR322 plasmid cleavage by: (**A**) **IrPCp** and **IrPNr**. Lanes: 1, plasmid−control; 2, plasmid + 50 μM **IrPCp**; 3, plasmid + 50 μM **IrPCp** + 50 μM H_2_O_2_; 4, plasmid + 50 μM **IrPCp** + 50 μM H_2_O_2_ + DMSO; 5, plasmid + 50 μM **IrPCp** + 50 μM H_2_O_2_ + 0.1 M NaN_3_; 6, plasmid + 50 μM **IrPCp** + 50 μM H_2_O_2_ + 0.62 μM SOD; 7, plasmid + 50 μM **IrPNr**; 8, plasmid + 50 μM **IrPNr** + 50 μM H_2_O_2_; 9, plasmid + 50 μM **IrPNr** + 50 μM H_2_O_2_ + DMSO; 10, plasmid + 50 μM **IrPNr** + 50μM H_2_O_2_ + 0.1 M NaN_3_; 11, plasmid + 50μM **IrPNr** + 50 μM H_2_O_2_ + 0.62 μM SOD; 12, plasmid + DMF; (**B**) by **IrPLm** and **IrPSf**. Lanes: 1, plasmid−control; 2, plasmid + 50 μM **IrPLm**; 3, plasmid + 50 μM **IrPLm** + 50 μM H_2_O_2_; 4, plasmid + 50 μM **IrPLm** + 50 μM H_2_O_2_ + DMSO; 5, plasmid + 50 μM **IrPLm** + 50 μM H_2_O_2_ + 0.1 M NaN_3_; 6, plasmid + 50 μM **IrPLm** + 50 μM H_2_O_2_ + 0.62 μM SOD; 7, plasmid + 50 μM **IrPSf**; 8, plasmid + 50 μM **IrPSf** + 50 μM H_2_O_2_; 9, plasmid + 50 μM **IrPSf** + 50 μM H_2_O_2_ + DMSO; 10, plasmid + 50 μM **IrPSf** + 50μM H_2_O_2_ + 0.1 M NaN_3_; 11, plasmid + 50μM **IrPSf** + 50 μM H_2_O_2_ + 0.62 μM SOD; 12, plasmid + DMF.

All complexes in presence of hydrogen peroxide caused distinct changes in the plasmid structure, resulting in increased amounts of the nicked form (Figure 5A lines 3, 8, and Figure 5B lines 3, 8). When the hydroxyl radical inhibitor—DMSO—was added to the reaction mixtures, evident inhibition of the DNA damage was observed, suggesting the involvement of hydroxyl radical in the cleavage process (Figure 5A lines 4, 9, and Figure 5B lines 4, 9). Furthermore, slight inhibition of the DNA cleavage was observed in the other cases viz., NaN_3_ (Figure 5A lines 5, 10, and Figure 5B lines 5, 10) and SOD (Figure 5A lines 6, 11, and Figure 5B lines 6, 11) validating the presence of singlet oxygen and superoxide anion radical, respectively.

Overall, the investigated complexes (**IrPCp**, **IrPNr**, **IrPLm**, **IrPSf**) are not presumably capable of double-strand plasmid damage leading to the formation of a linear form of plasmid (form III). Since it is well-known that cancer cells show obstinately high levels of intracellular ROS that can be involved in iridium-based redox reactions (Ir(III)/(IV) complexes), DNA oxidative damage by radicals cannot be excluded. However, any oxidative DNA damages are not desirable for any new chemotherapeutics, as they may contribute to overall genotoxicity [48]. The low level of damage of genetic material should be considered a privilege of these new complexes.

#### 2.2.2. Molecular Docking Study of the Interactions between DNA and Ir(III) Complexes

The binding poses obtained from docking simulations performed for each of the metal complexes studied herein were clustered with a 2 Å RMSD threshold. Considering that the absolute value of the difference in the lowest binding energy associated with any pair of clusters is below 2.8 kcal/mol, which is close to the standard deviation of AutoDock force field [52] employed for scoring, analysis of the results was essentially based on the cluster size. About 30 clusters of binding poses were obtained for particular ligands (28, 30, 27, and 31 for **IrPCp**, **IrPNr**, **IrPLm**, and **IrPSf**, respectively).

**Figure 6:**
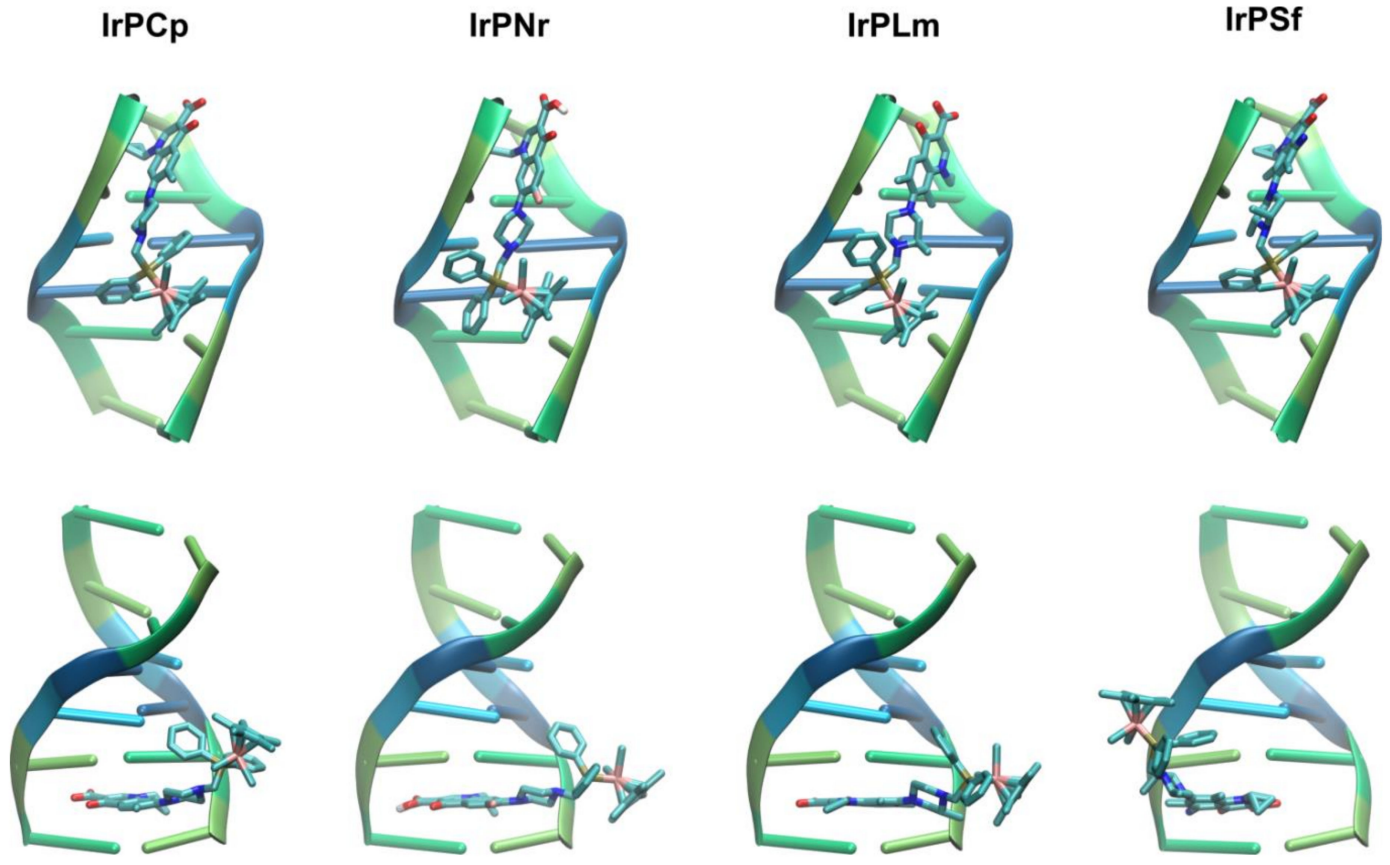
Representative structures of the binding modes of DNA-phosphinoiridium(III) complexes: top, minor groove binding; and bottom, threading intercalation.

Two binding modes were obtained overall, including minor groove binding and threading intercalation with the fluoroquinolone part of the molecule involved in pi stacking interactions and the Ir(III)-containing region positioned within the major or minor groove. The majority of the binding poses correspond to minor groove binding followed by intercalation coupled with major groove binding (Table 2). At most, 17% of the total number of binding poses exhibited threading intercalation accompanied by minor groove binding. The representative structures associated with both of the prevailing binding modes are presented in Figure 6.

Since numerous clusters featured a limited number of members, suggesting that a particular binding mode is significantly less likely, we performed a similar analysis using the clusters encompassing at least six members (Table 2). Focusing on the largest clusters further reinforced the conclusion of dominant minor groove binding and major groove binding-coupled intercalation, as no binding poses were found representing intercalation with the Ir(III) part of the complex positioned within the minor binding groove. Except for DNA-**IrPNr** complexes, minor groove binding appears to be predominant. In particular, the dual binding mode of intercalation associated with major groove binding might account for as little as 11% in the case of DNA-**IrPLm** complexes (Table 2). Interestingly, DNA-**IrPNr** complexes described in the most populated clusters appear to favor intercalation and major groove binding over the minor groove binding, which may be linked to the highest value of Ksv for intercalation of this particular ligand compared with the remaining phosphinoiridium(III) compounds (Table 1). It was concluded from the analysis of Ksv values that major groove binding is also present in the complexes studied herein, albeit to a modest extent. While no major groove binding was observed in the docking results, the positioning of the Ir(III) part of the intercalated ligands in the major binding groove may explain the experimental findings. Overall, the docking results appear to be in agreement with the Ksv values, supporting multimodal binding of Ir(III) complexes with the prevalence of minor groove binding.

### 2.3. Possible Reaction with Proteins

#### 2.3.1. Human Serum Albumin Interaction Study

**Figure 7:**
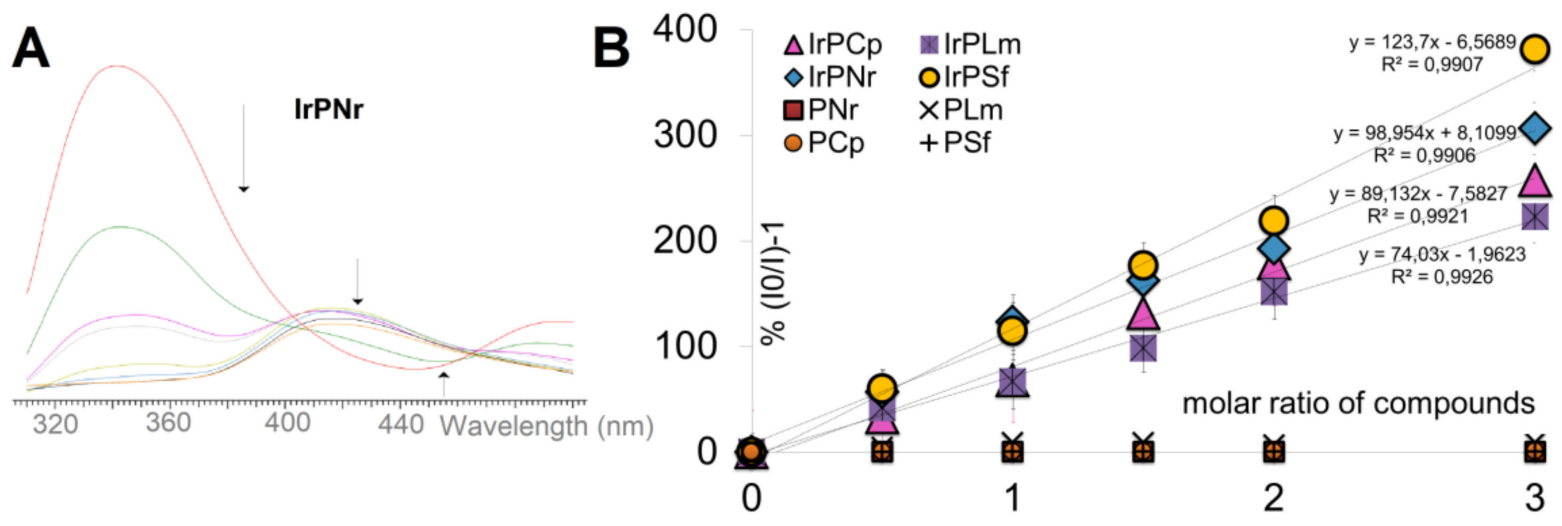
(**A**) Fluorescence quenching of HSA (C = 5 × 10^−5^ M) by **IrPNr**, (molar ratios 0.5, 1, 1.5, 2, 3, 4, 5, 6) in 50 mM pH 7.4 phosphate buffer; (**B**) Stern–Volmer plots of HSA (C = 10 μM) quenching by **PCp**, **PNr**, **PSf**, **PLm**, **IrPCp**, **IrPNr**, **IrPSf**, **IrPLm** (I_0_ and I—intensity of HSA in the absence and presence of the increasing amounts of the compounds, respectively).

Interest in studying the interaction of metal ions and their compounds with blood plasma proteins, especially with serum albumin (HSA), the most abundant protein in plasma involved in the transport of drugs through the bloodstream, is increasing [27,53]. This molecule has two main binding sites: site I is located in subdomain IIA and site II is located in subdomain IIIA. However, only the first binding site contains tryptophan residue that possesses intrinsic fluorescence—Trp-214 residue (with an excitation wavelength of 295 nm) [54,55]. Changes in the appearance of the Trp-214 residue emission band in HSA, in the presence of the tested compounds, may indicate changes in the protein’s conformation, the association of its subunits or denaturation, or the binding of the compound to protein [53,56]. For all the tested compounds (except phosphine ligands) with an increase of concentrations, the intensity of the HSA emission band at 342 nm gradually decreased with the simultaneous appearance of an additional fluorescence band with an emission maximum at 425 nm (Figure 7, Supplementary Figure S5) that can be assigned to the fluorescence from the starting complexes or new system HSA-Ir(III) complex.

Changes in the intensity and shift of the band maximum toward longer wavelengths indicate the strong interaction of each complex with HSA, increased polarization in the area surrounding the tryptophan residue and/or the energy transfer between the tested compounds and HSA [13]. The strength of albumin quenching can be presented in the following order: **IrPSf** > **IrPLm** > **IrPCp** > **IrPNr** (Figure 7, Ksv values, Table 3). This phenomenon can be related to the presence of additional fluorine atoms in the quinolone moiety and methyl group attached to the piperazine ring. Furthermore, the affinity of HSA for all the considered Ir(III) complexes with fluoroquinolone derivatives is much higher than for copper(I) complexes with the same organic derivates [11,13,41,42,43]. It is noteworthy that the tendency of the strength of HSA quenching is different from observations for all complexes (Cu(I) and Ru(II)) with the same phosphines derived from fluoroquinolones [11,41,42,43,44].

#### 2.3.2. Molecular Docking Study of the Interactions between Human Albumin and Ir(III) Complexes

The largest clusters obtained from the docking of particular Ir(III) complexes either correspond to the lowest binding energy structures (59 and 33-member clusters for **IrPNr** and **IrPLm**, respectively) or differ by at most 0.5 kcal/mol from the lowest binding energy observed within a given cluster (27 and 37 member clusters for **IrPCp** and **IrPSf**, respectively). Considering the significant difference in the size between the first and remaining clusters for each of the compounds studied herein (i.e., the size of the second largest cluster constituting about 56, 27, 21, and 32% of the number of members in the first largest cluster of **IrPCp**, **IrPNr**, **IrPLm**, and **IrPSf**, respectively), the most probable binding mode was associated with the representative structure of the largest cluster.

**Figure 8:**
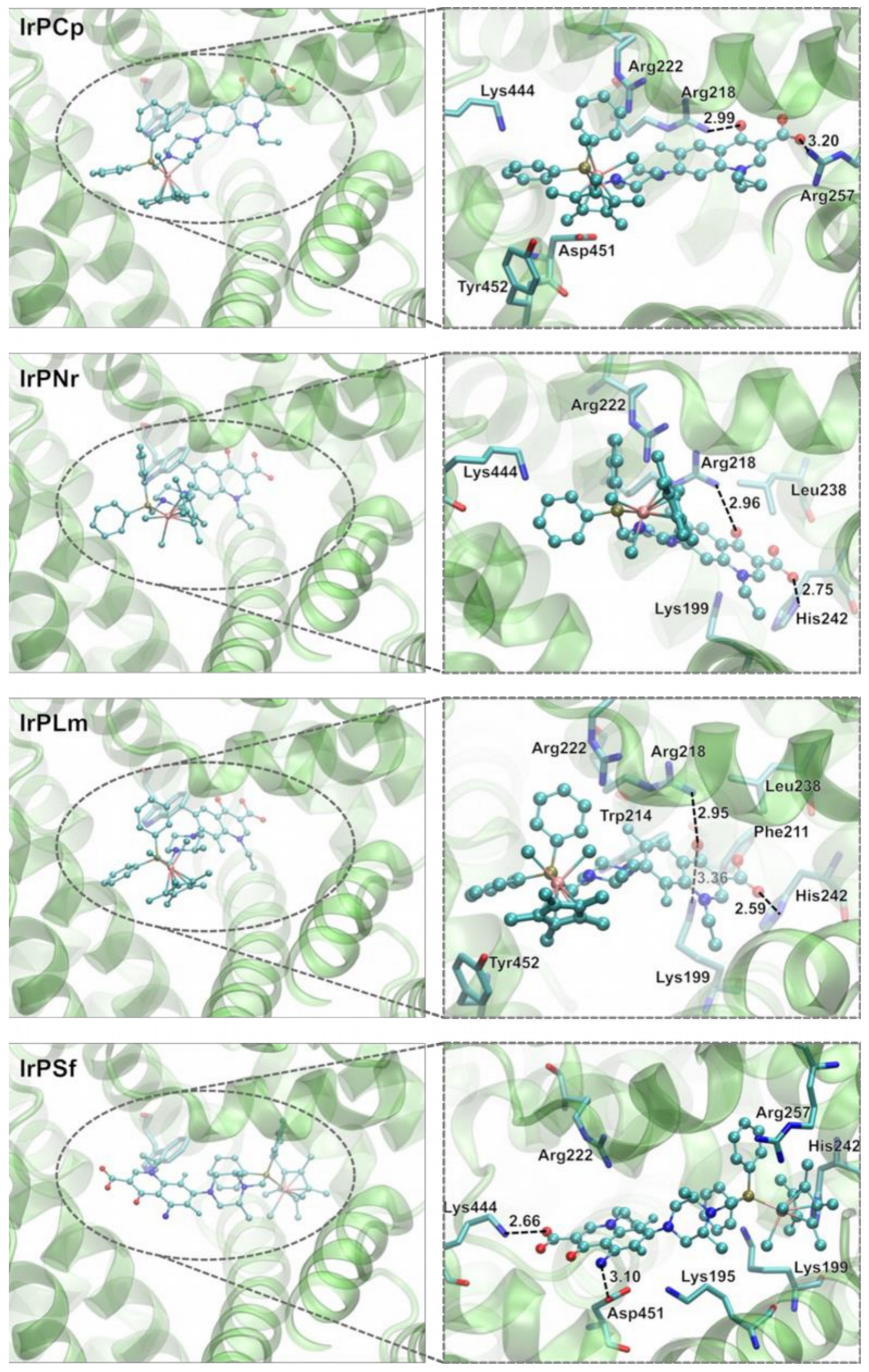
Binding mode of HSA-phosphinoiridium(III) complexes. The docked compounds are shown in ball-and-stick representation. Trp214 residue is shown in stick representation in the panels on the left side. In the right side panels are all the HSA residues within 3 Å of the metal complexes. The selected distances are given in Å. Hydrogen atoms are not shown for clarity.

The binding mode of particular complexes is presented in Figure 8. All the compounds considered herein were found to bind up to 5 Å of Trp214 residues. In the case of **IrPLm**, **IrPNr**, and **IrPSf,** the shortest distance to Trp214 is equal to 2.91, 3.24, and 3.54 Å, respectively, whereas **IrPCp**-Tyr214 separation amounts to 4.94 Å. Presumably, such a distance is short enough to affect the experimentally observed fluorescence quenching of Trp214. Despite the longer distance to Trp214 residue characterizing the **IrPCp** complex, all four complexes appear to occupy the same region of the binding site located in HSA subdomain IIA (see the left side panel in Figure 8).

**IrPCp**, **IrPNr**, and **IrPLm** feature similar binding modes with the Ir(III) core located at the entrance of the binding pocket and fluoroquinolone part buried deeply in the binding site. This binding mode facilitates the formation of at least two hydrogen bonds. In particular, the hydrogen bond between the oxygen atom present in the quinolone moiety and Arg218 residue was observed in all three complexes (see the right- side panel in Figure 8 for a close-up view of interactions). Another hydrogen bond might occur between the compound carboxylate group and His242 (**IrPNr** and **IrPLm**) or Arg257 residue (**IrPCp**). In the case of the **IrPNr** complex, the quinolone oxygen atom could also be hydrogen-bonded to Lys199 residue. Possible hydrophobic interactions may occur between the phenyl ring of phosphine ligand in **IrPCp**, **IrPNr**, **IrPLm** complexes and Pro447, Cys448, and Cys437 residues.

Unlike the three complexes discussed above, **IrPSf** binds in the opposite way, i.e., with the Ir(III) part buried in the binding pocket. Accordingly, the hydrogen-bonding pattern observed in **IrPCp**, **IrPNr**, and **IrPLm** complexes are no longer possible, as the molecule region surrounding Ir(III) core is essentially nonpolar. However, the fluoroquinolone moiety can still be engaged in hydrogen-bonding with Lys444 and Asp451 residues. The nonpolar cyclopentadienyl ligand is surrounded by the hydrophobic patch of Cys200, Cys245, Cys246, and Cys253 residues.

Despite the presumably different binding mode of **IrPSf**, a similar number of interactions characterizing binding of all the iridium complexes analyzed herein appears to be in line with the experimental results demonstrating minor differences in the binding strength of these compounds. Moreover, the distinct binding mode of the **IrPSf** complex might account for the strongest binding of this particular compound, as shown in the fluorescence quenching experiment.

#### 2.3.3. Apo-Tranferrin Interaction Study

Interactions between Ir(III) complexes and their parent fluoroquinolones-based ligands with the second selected transporter protein, apo-transferrin (apo-Tf), were studied in detail. The protein apo-Tf has been recently considered and investigated as a possible transporter of metal complexes [57].

**Figure 9:**
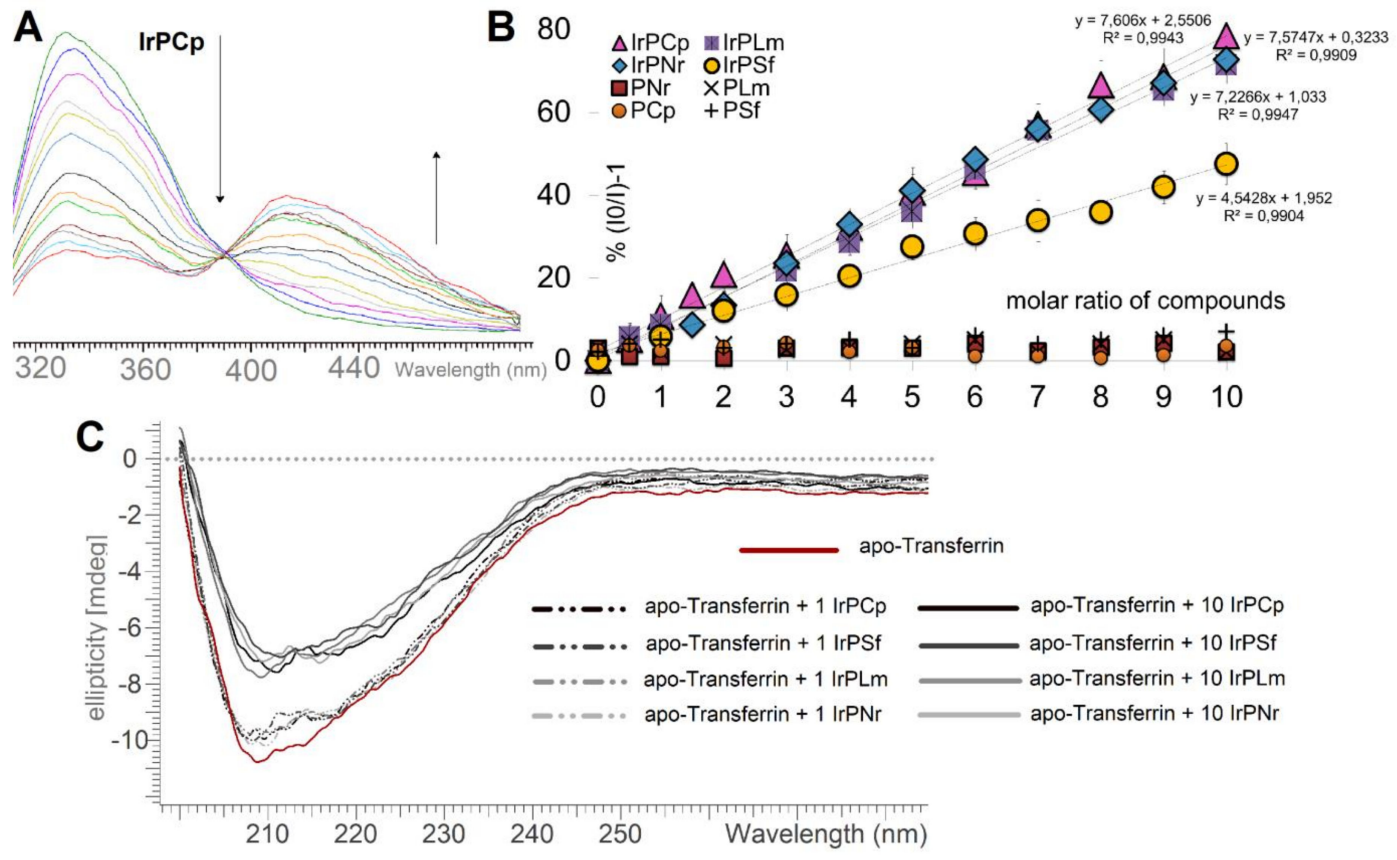
(**A**) Fluorescence quenching of apo-Tf (C = 3.6 × 10^−6^ M) by IrPCp (molar ratios 0.5, 1, 1.5, 2, 3, 4, 5, 6, 7, 8, 9, 10) in 50 mM pH 7.4 phosphate buffer; (**B**) Stern–Volmer plots of apo-Tf (C = 3.6 μM) quenching by **IrPCp**, **IrPNr**, **IrPSf**, **IrPLm**, **PCp**, **PNr**, **PSf**, **PLm** (I_0_ and I—intensity of HSA in the absence and presence of the increasing amounts of the compounds, respectively); (**C**) Circular dichroism spectra of apo-transferrin with Ir(III) complexes.

A solution of apo-Tf was titrated with increasing concentrations of all the tested compounds, and the fluorescence intensity of apo-Tf was monitored spectrophotometrically (Figure 9A; Supplementary Materials, Figures S6 and S7). At the same time, Ksv values were determined from Stern–Volmer plots (Figure 9B, Table 4). The fluorescence intensity of apo-Tf at 343 nm decreased gradually, suggesting that investigated compounds can penetrate the apo-transferrin structure and interrupt the tryptophan’s microenvironment.

Furthermore, as depicted in Table 4, the Ksv for all complexes ranged from 1.32 × 10^4^ to 2.18 × 10^4^ M^−1^. Thus the interaction between Ir(III) complexes and apo-Tf follows a static quenching mechanism. The strength of apo-Tf fluorescence quenching by complexes can be presented in the following order: **IrPCp** > **IrPNr** > **IrPLm** > **IrPSf**. Among all studied complexes, **IrPSf** was the least efficient in interactions with apo-Tf; this can be explained by the most extensively substituted structure of sparfloxacin, which contains the largest number of substituents among all studied antibiotics. In-depth analysis of Stern–Volmer plots leads to conclusion that from the perspective of more efficient binding to apo-Tf, the absence of the methyl group substituent in the piperazine motif plays a crucial role. Moreover, the complexes interact with apo-transferrin more strongly than organic derivatives.

In order to monitor Ir(III) complex binding, CD spectra of apo-transferrin were recorded (Figure 9C) where three regions—visible (320–600 nm), aromatic (230–320 nm), and intrinsic (180–260 nm)—deliver different information about protein structure [58]. The bands in the CD spectrum of apo-transferrin in the 230–320 nm (aromatic) range are attributed to the optical activity of tyrosine and tryptophan residues. As in HSA, the dichroism in the intrinsic region (180–260 nm) of apo-transferrin is related to secondary structure [59]. Changes in this region of the spectrum when the protein is incubated with metal complexes indicate that the drugs affect the conformation. The presented results (Figure 9C) indicate that a 10-fold excess of the Ir(III) complexes did not alter the helical structure of the protein. These results may be of great importance since transferrin needs to be recognized by specific receptors to be delivered into the cells. Given that two tyrosine residues are directly involved in the binding of iron, and tryptophan residue is in close proximity [31,32], it is reasonable to assume that the studied Ir(III) complexes are binding at the specific binding sites for Fe^3+^ ions (see Figure 9C).

Remarkably, the interactions of Ir(III) complexes with studied proteins revealed a diverse mode of the fluorescence quenching, as determined orders of quenching altered differently—HSA: **IrPSf** > **IrPLm** > **IrPCp** > **IrPNr**; apo-Tf: **IrPCp** > **IrPNr** > **IrPLm** > **IrPSf**. This diverse mode suggests that different parts of the complexes and different interactions are probably responsible for the emission quenching of HSA and apo-Tf.

#### 2.3.4. Molecular Docking Study of the Interactions between Apo-Transferrin and Ir(III) Complexes

**Figure 10:**
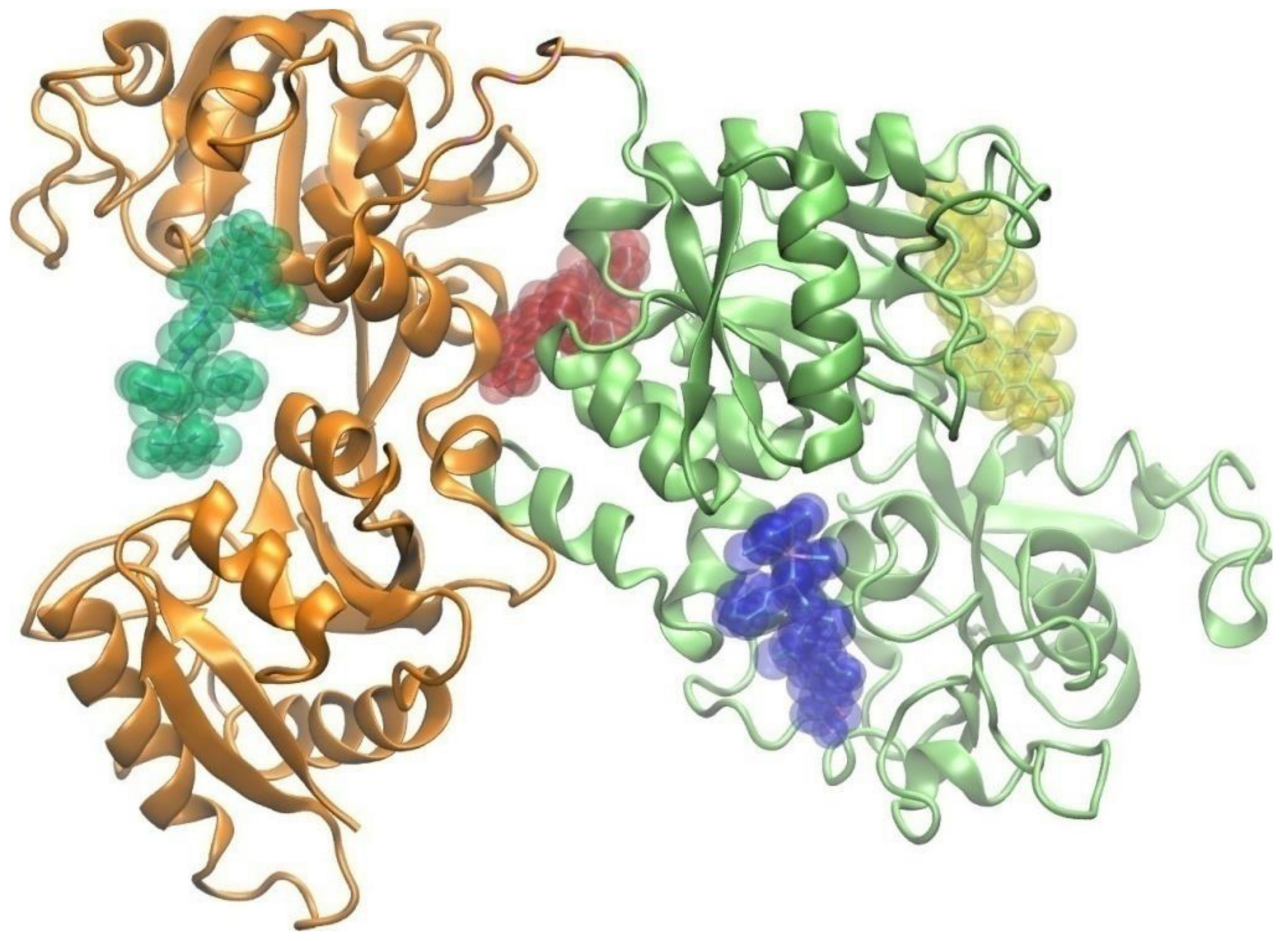
The predicted binding pockets within apo-Tf structure. The N- and C-lobes of apo-Tf are shown in orange and light green colors. The location of #1, #2, #3, and #4 binding sites is specified by the docked **IrPCp** shown in blue, red, yellow, and dark green colors, respectively.

All the predictions regarding possible apo-Tf binding pockets yielded relatively consistent results encompassing four binding sites presented in Figure 10. In particular, binding pockets #1 and #3 correspond to iron-binding sites located in C- and N-lobes of apo-Tf, and the #2 binding pocket is positioned at the interface between the two lobes, whereas the #4 binding pocket is located in the proximity of the C-lobe iron-binding site. Remarkably, all four binding pockets involve nearby tyrosine or tryptophan residues that can be disturbed by binding of the metal complex.

Ir(III) complexes were found to dock within all four binding sites considered herein. The lowest interaction of energy characterized binding poses was determined for the #2 pocket. All the compounds docked in this particular binding site consistently yielded a lower binding energy value by approximately 3 kcal/mol compared to the binding poses established for the remaining binding pockets. This fact suggests that the binding of iridium(III) complexes is the most favorable in the case of the #2 pocket, but other binding modes are also possible. Considering that the binding energy difference between the #2 pocket and the remaining predicted binding sites exceed the standard deviation of AutoDock force field [52] used for ranking the complexes, #2 pocket binding modes will be discussed in further detail. By pursuing the same reasoning as the HSA docking results, the most plausible binding poses were assumed to be associated with the largest cluster obtained for particular Ir(III) complexes.

Noticeably, the largest clusters were either found to feature the lowest binding energy (27 and 31 member clusters of **IrPNr** and **IrPSf** compounds, respectively) or to differ by 1.7 kcal/mol at most from the lowest energy cluster (which is well within the 2.5 kcal/mol standard deviation of AutoDock force field [52]), as observed in the case of the 34 member cluster of **IrPLm**. The largest cluster obtained of the apo-Tf-**IrPCp** complex consisted of 31 members, closely followed by the second largest cluster at 25 members, featuring a similar binding mode and energy. The difference in the binding energy that characterizes these two clusters and the lowest energy cluster obtained for the apo-Tf-**IrPCp** complex does not exceed 1 kcal/mol, further justifying the choice of this particular binding mode.

**Figure 11:**
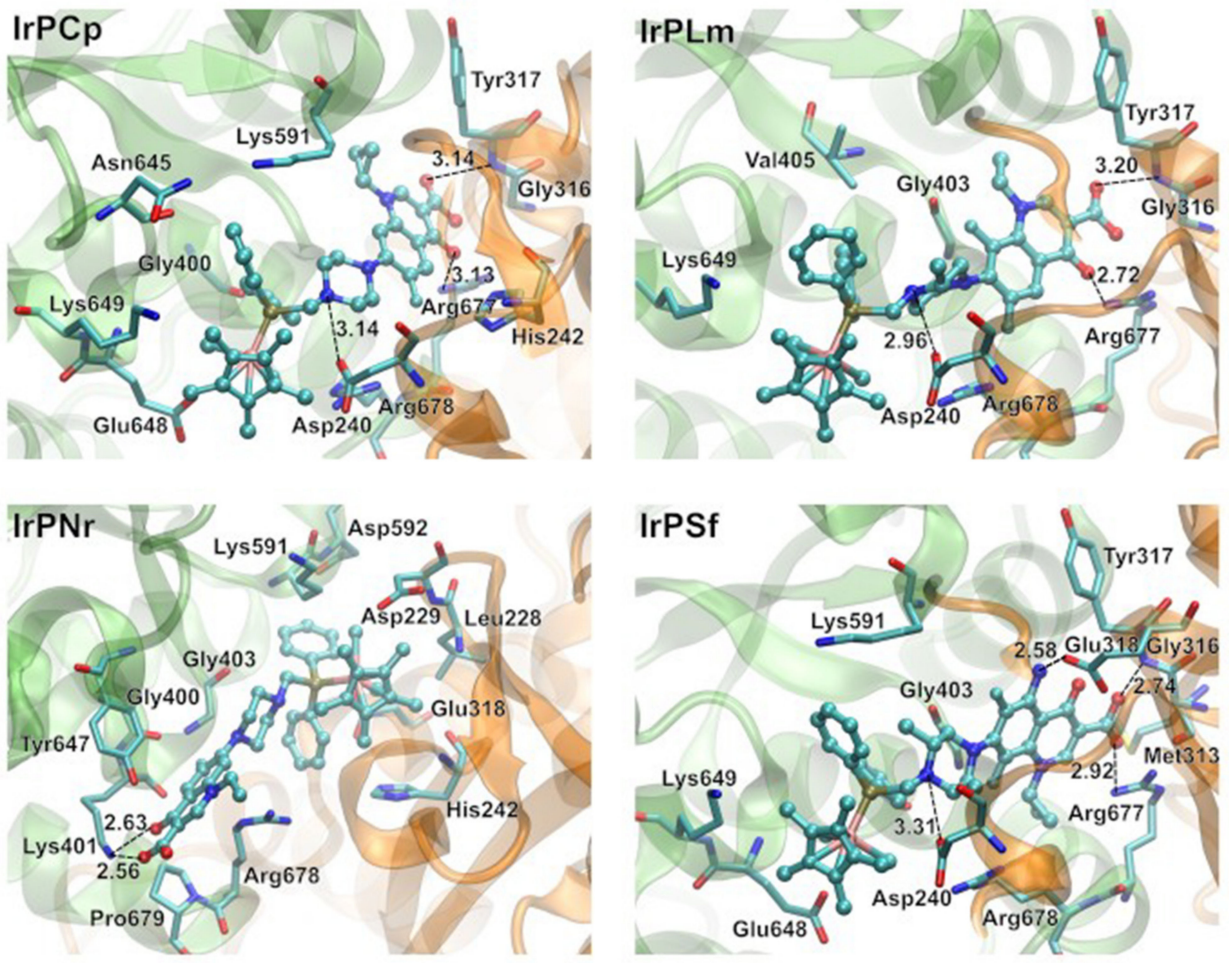
Binding mode of apo-Tf-complexes within the #2 binding pocket. The docked compound’s molecules are shown in ball-and-stick representation. All the apo-Tf residues within 3 Å of the metal complexes are presented in stick representation. The selected distances are given in Å. Hydrogen atoms are not shown for clarity.

The representative structures of the largest clusters obtained for iridium(III) complexes occupying the #2 binding pocket are demonstrated in Figure 11. All the docked compounds are wedged between Tyr317 and Tyr647 residues. In the prevalent binding mode featured by **IrPCp**, **IrPLm**, and **IrPSf** compounds, the shortest distances between fluoroquinolone moiety and Tyr317 residue are equal to 2.11, 2.00, and 1.77 Å, respectively. The shortest separation between the metal core of these complexes and Tyr647 residue is within the 3.2–3.8 Å range. **IrPNr** complex is oppositely positioned to the remaining compounds, with the fluoroquinolone part-Tyr647 distance of 2.00 Å and the metal core separated by 3.46 Å from Tyr317 residue. At least three hydrogen bonds can be formed in the binding mode displayed by **IrPCp**, **IrPLm**, and **IrPSf**. The oxygen atoms present in the fluoroquinolone moiety are prone to hydrogen-bonding interactions with the side chain of Arg677 and the backbone nitrogen atom of Tyr317 residues, whereas Asp240 residue is positioned close enough to certain nitrogen atoms of a piperazine ring, facilitating the formation of another hydrogen bond. The amino group of the **IrPSf** fluoroquinolone part might engage in an additional hydrogen bond with Glu318 residue (see Figure 11). The hydrophobic interactions of **IrPCp**, **IrPLm**, and **IrPSf** compounds may be mediated by Cys402, Leu404, Val405, Met382, and Cys674 residues positioned within 4 Å of particular metal complexes. The distinct binding mode of **IrPNr** features two possible hydrogen-bonding interactions between oxygen- bearing fluoroquinolone moiety and Lys401 residue (Figure 11). Nonpolar residues close enough to participate in hydrophobic interactions with **IrPNr** include Cys227, Leu228, and Cys241 residues.

Overall, the binding mode of the representative structures from the largest clusters of the #2 binding pocket might indicate the strongest binding of **IrPSf** featuring an additional hydrogen bond to amino substituent at the fluoroquinolone aromatic rings. Although this conclusion is not exactly supported by the experimental data demonstrating the weakest binding of **IrPSf** by apo-Tf (Table 4), it should be kept in mind that the possible multiple binding mode of iridium(III) complexes (i.e., engaging other binding pockets) cannot be excluded based on the current results and may affect the general binding characteristics.

## 3. Materials and Methods

### 3.1. Materials

Calf thymus DNA (CT DNA), human serum albumin (HSA), apo-transferrin (apo-Tf), pBR322 DNA, sodium azide (NaN_3_), DMSO, superoxide dismutase (SOD), methyl green, DAPI, ethidium bromide (EB), and other chemicals and solvents were purchased from Sigma–Aldrich (Hamburg, Germany). All solvents were deaerated before use.

### 3.2. Methods

#### 3.2.1. Physical Measurements

Single crystals of **IrPCp**·CHCl_3_, **IrPLm**·1.5CHCl_3_, and **IrPNr**·2CHCl_3_ were collected on a SuperNova diffractometer using graphite monochromatic MoK_α_ radiation at 293 K, 100 K, or 293 K, respectively. Data processing was undertaken with CrysAlisPRO (Yarnton, England) The structures were solved using direct methods, and for refinement, the non-H atoms were treated anisotropically. The main calculations were performed with SHELXL. Crystallographic data of the structures was deposited at the Cambridge Crystallographic Data Centre with CCDC reference numbers 1996074 (**IrPCp**·CHCl_3_), 1996071 (**IrPLm**·1.5CHCl_3_), and 1996073 (**IrPNr**·2CHCl_3_).

Elemental analyses (C, H, and N) were conducted with a Vario MICRO Cube (Elementar, Auckland, New Zealand). NMR spectra were recorded using a Bruker Avance II 300 MHz spectrometer in CDCl_3_ with traces of CHCl_3_ as an internal reference for ^1^H and 85% H_3_PO_4_ in H_2_O as an external standard for ^31^P{^1^H}. Mass spectra were recorded with a Bruker MicrOTOF-Q II spectrometer with an ESI ion source under the following nebulizer pressure conditions: 0.4 bar, dry gas: 4.0 l min^−1^ heated to 180 °C. Data were recorded in the positive ion mode, while profile spectra were recorded in the mass range of 50–3000 *m*/*z*; end plate offset—500 V; capillary voltage 4500 V; mass resolving power of the instrument—over 18,000. Mass calibration was done using the cluster method with a mixture of 10 mM sodium formate and isopropanol (1:1, *v*/*v*) before the run [14].

#### 3.2.2. Characterization of Organometallic Iridium(III) Complexes

All iridium(III) complexes (**IrPCp**, **IrPNr**, **IrPLm**, **IrPSf**) were synthesized according to the literature procedure described by our group [14].

Data for [Ir(η^5^-Cp*)Cl_2_**PCp**] (IrPCp)

**Yield:** 80%. Anal. found: C, 47.01; H, 4.32; N, 4.00%. Anal. calc. for C_40_H_44_Cl_2_FIrN_3_O_3_P·CHCl_3_ (C_41_H_45_Cl_5_FIrN_3_O_3_P): C, 47.02; H, 4.33; N, 4.01%.

**NMR (298 K, CDCl_3_):^31^P{^1^H}:** −1.30 (P^1^, s); **^1^H:** 1.11–1.33 (H^72,73^, m, 4-H), 1.35 (H^1^, d, 2.2 Hz, 15-H), 2.39 (H^13,14^, bt, 4.8 Hz, 4-H), 2.99 (H^12,15^, bt, 4.8 Hz, 4-H), 3.47 (H^71^, m, 1-H), 4.07 (H^11^, s, 2-H), 7.16 (H^69^, d, 7.1 Hz, 1-H), 7.44–7.53 (H^43,44^, m, 6-H), 7.92 (H^63^, d, 13.3 Hz, 1-H), 8.00–8.11 (H^42^, m, 4-H), 8.72 (H^67^, s, 1-H), 15.02 (H^70^, s, 1-H).

**^+^ESI-MS (CHCl_3_/H_2_O, *m*/*z*):** 928.21 [M + H]^+^, 892.23 [M − Cl]^+^, 888.28 [M − 2Cl + CH_3_OH]^+^, 856.26 [M − 2Cl]^+^.

Crystals of **IrPCp**·CHCl_3_ suitable for X-ray analysis were obtained under refrigeration by slow diffusion of hexane into a solution of the complex in chloroform under normal oxygen conditions. Crystal data: C_41_H_45_Cl_5_FIrN_3_O_3_P, M = 1047.22 g mol^−1^, crystal size: 0.20 × 0.20 × 0.02 mm; crystal system: triclinic, space group: P1, a = 9.0544(4) Å, b = 11.8197(5) Å, c = 20.8951(8) Å, α = 85.841(3)°, β = 86.837(3)°, γ = 67.930(4)°, V = 2065.94(16) Å^3^, D_calc_ (Z = 2) = 1.683 g cm^−3^, θ range for data collection: 4.042 to 71.700°, Mo Kα radiation (λ = 1.54184 Å), μ_Mo_ = 9.983 mm^−1^, reflections collected/unique: 28 428/7969, [R_int_ = 0.0886], completeness to θ full = 99.9%, final R indices [I > 2σ(I)]: R_1_ = 0.0440, wR_2_ = 0.1081, R indices (all data): R_1_ = 0.0544, wR_2_ = 0.1169, GOF = 1.026, largest diff. peak and hole: 1.919 and −1.931 e Å^−3^, data/restraints/parameters: 7969/0/504, T = 293 K.

Data for [Ir(η^5^-Cp*)Cl_2_**PSf**] (**IrPSf**)

**Yield:** 80%. Anal. found: C, 50.97; H, 4.87; N, 5.65%. Anal. calc. for C_42_H_48_Cl_2_F_2_IrN_4_O_3_P: C, 51.01; H, 4.89; N, 5.67%.

**NMR (298 K, CDCl_3_):^31^P{^1^H}:** −3.06 (P^1^, s); **^1^H:** 0.72 (H^16,17^, d, 6.4 Hz, 6-H), 1.04–1.20 (H^72,73^, m, 4-H), 1.33 (H^1^, d, 2,2 Hz, 15-H), 2.45 (H^12,15^, m, 2-H), 2.80 (H^13,14^, m, 2-H), 3.07 (H^13,14^, m, 2-H), 3.87 (H^71^, m, 1-H), 4.25 (H^11^, s, 2-H), 6.41 (H^63^, bs, 2-H), 7.43–7.51 (H^43,44^, m, 6-H), 7.98–8.13 (H^42^, m, 4-H), 8.61 (H^67^, s, 1-H), 14.65 (H^70^, bs, 1-H).

**^+^ESI-MS (CHCl_3_/H_2_O, *m*/*z*):** 989.25 [M + H]^+^, 949.32 [M − 2Cl + CH_3_OH]^+^, 919.32 [M − 2Cl]^+^.

Data for [Ir(η^5^-Cp*)Cl_2_**PLm**] (**IrPLm**)

**Yield:** 80%. Anal. found: C, 44.20; H, 4.15; N, 3.72%. Anal. calc. for C_40_H_45_Cl_2_F_2_IrN_3_O_3_P·1.5CHCl_3_ (C_41.5_H_46.5_Cl_6.5_F_2_IrN_3_O_3_P): C, 44.23; H, 4.16; N, 3.73%.

NMR (298 K, CDCl_3_): **^31^P{^1^H}:** −1.82 (P^1^, s); **^1^H:** 0.62 (H^16^, d, 6.3 Hz, 3-H), 1.34 (H^1^, d, 2.2 Hz, 15-H), 1.50 (H^72^, t, 7.0 Hz, 3-H), 2.14–3.14 (H^12,13,14,15^, m, 7-H), 4.15 (H^11^, dd, J_1_ = 40 Hz, J_2_ = 16 Hz, 2-H), 4.40 (H^71^, qd, J_1_ = 7.2 Hz, J_2_ = 3.3 Hz, 2-H), 7.39–7.56 (H^43,44^, m, 6-H), 7.87 (H^63^, dd, J_1_ = 12.0 Hz, J_2_ = 1.7 Hz, 1-H), 7.99–8.15 (H^42^, m, 4-H), 8.55 (H^67^, s, 1-H), 14.68 (H^70^, s, 1-H).

**^+^ESI-MS (CHCl_3_/H_2_O, *m*/*z*):** 948.22 [M + H]^+^, 914.26 [M − Cl]^+^, 906.28 [M − 2Cl + CH_3_OH]^+^, 878.28 [M − 2Cl]^+^.

Crystals of **IrPLm**·1.5CHCl_3_ suitable for X-ray analysis were obtained under refrigeration by slow evaporation of chloroform/methanol (1:1, *v*/*v*) solution under normal oxygen conditions. Crystal data: C_41.5_H_46.5_Cl_6.5_F_2_IrN_3_O_3_P, M = 1126.91 g mol^−1^, crystal size: 0.100 × 0.100 × 0.050 mm, crystal system: monoclinic, space group: I2/a, a = 24.6249(3) Å, b = 8.35470(1) Å, c = 44.3459(6) Å, α = 90°, β = 104.3440(10)°, γ = 90°, V = 8839.03(18) Å^3^, D_calc_ (Z = 8) = 1.694 g cm^−3^, θ range for data collection: 2.215 to 31.772°, Mo Kα radiation (λ = 0.71073 Å), μ_Mo_ = 3.501 mm^−1^, reflections collected/unique: 154 041/13 616, [R_int_ = 0.0395], completeness to θ full = 99.9%, final R indices [I > 2σ(I)]: R_1_ = 0.0321, wR_2_ = 0.0716, R indices (all data): R_1_ = 0.00.0394, wR_2_ = 0.0734, GOF = 1.059, largest diff. peak and hole: 3.325 and −0.922 e Å^−3^, data/restraints/parameters: 13 616/3/541, T = 100 K.

Data for [Ir(η^5^-Cp*)Cl_2_**PNr**] (**IrPNr**)

**Yield:** 80%. Anal. found: C, 42.60; H, 4.01; N, 3.63%. Anal. calc. for C_39_H_44_Cl_2_FIrN_3_O_3_P·2CHCl_3_ (C_41_H_46_Cl_8_FIrN_3_O_3_P): C, 42.65; H, 4.02; N, 3.64%.

NMR (298 K, CDCl_3_):**^31^P{^1^H}:** −1.38 (P^1^, s); **^1^H:** 1.35 (H^1^, d, 2.2 Hz, 15-H), 1.54 (H^72^, t, 7.3 Hz, 3-H), 2.39 (H^13,14^, bt, 4,6 Hz 4-H), 2.97 (H^12,15^, bt, 4,6 Hz, 4-H), 4.07 (H^11^, s, 2-H), 4.25 (H^71^, q, 7.2 Hz, 2-H), 6.64 (H^69^, d, 6.9 Hz, 1-H), 7.42–7.54 (H^43,44^, m, 6-H), 7.96 (H^63^, d, 13.2 Hz, 1-H), 8.00–8.11 (H^42^, m, 4-H), 8.62 (H^67^, s, 1-H), 15.09 (H^70^, s, 1-H).

**^+^ESI-MS (CHCl_3_/H_2_O, *m*/*z*):** 916.21 [M + H]^+^, 980.24 [M − Cl]^+^, 876.29 [M − 2Cl + CH_3_OH]^+^, 844.27 [M − 2Cl]^+^.

Crystals of **IrPNr**·2CHCl_3_ suitable for X-ray analysis were obtained under refrigeration by slow evaporation of chloroform/acetone (1:1, *v*/*v*) solution under normal oxygen conditions. Crystal data: C_41_H_46_Cl_8_FIrN_3_O_3_P, M = 1154.58 g mol^−1^, crystal size: 0.300 × 0.300 × 0.200 mm, crystal system: monoclinic, space group: P2_1_/n, a = 10.1356(2) Å, b = 17.3727(3) Å, c = 25.6237(4) Å, α = 90°, β = 93.137(1)°, γ = 90°, V = 4505.13(14) Å^3^, D_calc_ (Z = 4) = 1.702 g cm^−3^, θ range for data collection: 3.075 to 71.674°, Mo Kα radiation (λ = 1.54184 Å), μ_Mo_ = 10.818 mm^−1^, reflections collected/unique: 69 550/8730, [R_int_ = 0.1085], completeness to θ full = 99.8%, final R indices [I > 2σ(I)]: R_1_ = 0.0642, wR_2_ = 0.1514, R indices (all data): R_1_ = 0.00710, wR_2_ = 0.1560, GOF = 1.108, largest diff. peak and hole: 2.658 and −2.947 e Å^−3^, data/restraints/parameters: 8730/0/533, T = 293 K.

#### 3.2.3. Interaction with Calf Thymus DNA

The stock solution was prepared by dissolving of the calf thymus (CT DNA) in 50 mM phosphate buffer saline (PBS) (pH = 7.4). The CT DNA concentration (C = 5 × 10^−5^ M) was determined by a UV spectrophotometer using the molar absorption coefficient 6600 M^−1^m^−1^ at 258 nm [60]. The stock solution was stored at 4 °C and used for > 6 days. The luminescent complexes of CT DNA with EB (ethidium bromide), DAPI (4′,6-diamidyno−2-fenyloindol), and MG (methyl green) were prepared by mixing the substrates in the equimolar ratio (C_CT DNA-fluorescent dye_ = 5 × 10^−5^ M; 50 mM of the phosphate buffer at pH = 7.4). The solution of the CT DNA fluorescent dye system was titrated in different molar ratios (0.5, 1, 2, 3, 4, 5, 6, 7, 8, 9, and 10) with the tested substances (dissolved in DMSO) and incubated for 1 h for each portion of the investigated compounds. The final concentration of DMSO was 2% in each sample. Photoluminescence measurements were recorded at 298 K using a Cary Eclipse Fluorescence Spectrophotometer. The excitation wavelength was 510 nm for the CT DNA-EB complex, 358 nm for the CT DNA-DAPI complex, and 633 nm for the CT DNA-MG complex. The Inner Filter Effect was considered and the corrected intensity of the emission band was calculated using Equation (1) [13], where: Abs—absorbance at analyzed emission wavelength; I_f_—uncorrected emission intensity.

(1) $$I_{\mathrm{cor}}=\frac{I_{f}\;2.303\;Abs}{\;\left(1-10^{Abs}\right)}$$

In order to confirm the quenching mechanism, the fluorescence quenching was analyzed according to Stern–Volmer in Equation (2):

(2) $$\frac{I_{0}}{I}=1+K_{sv}\left[C\right]$$

where I_0_ and I are the fluorescence intensities in the absence and presence of the quencher, respectively, K_SV_ is the Stern–Volmer quenching constant, and [C] is the concentration of the quencher, respectively.

In same case, it was clearly observed that both static and dynamic quenching occur simultaneously where a nonlinear curve is observed. In such cases, extended Stern–Volmer plots can be used to describe the new situation with the following Equation (3) [61,62,63].

(3) $$\frac{1-(I/I_{0})}{\left[C\right]}=K_{DSV}(I/I_{0})\;+V$$

where I_0_ and I are the fluorescence intensities in the absence and presence of the quencher, respectively; K_DSV_ is the dynamic quenching constant; [C] is the concentration of the quencher, and V is the volume of the sphere of action. We drew the plots of ([1 − (I/I_0_)]/[C]) versus I/I_0_. K_DSV_ is the slope of the plot and the static quenching constant V is calculated from the intercept of the plot.

The spectra of circular dichroism were recorded using a spectropolarimeter JASCO J-715 (CD and MCD).

#### 3.2.4. DNA Strand Break Analysis

The ability of IrPCp, IrPNr, IrPSf, IrPLm, and phosphine ligands to induce single- or double-strand breaks in plasmid DNA was tested with the pBR322 plasmid (C = 0.5 mg/mL). All compounds were dissolved in DMF; the concentration was kept constant (10% by volume) in the final solution with/without hydrogen peroxide (H_2_O_2_, C = 50 μM), superoxide dismutase (SOD, C = 0.62 μM), dimethyl sulfoxide (DMSO, C = 1.4 mM), or sodium azide (NaN_3_, C = 40 mM) [64]. The reaction was also monitored after the addition of various groove binders: methyl green (MG, C = 10 mg/mL), and 4′,6-diamidino-2-phenylindole (DAPI, C = 5 mg/mL) [48,65,66]. After incubation for 1 h at 37°C, reaction mixtures (20 μL) were mixed with 3 μL of loading buffer (bromophenol blue in 30% glycerol) and loaded on 1% agarose gels, containing EB, in TBE buffer (90 Mm Tris–borate, 20 mM EDTA, pH = 8.0). Gel electrophoresis was performed at a constant voltage of 100V (4 Vcm-1) for 60 min. The gel was photographed and processed with a Digital Imaging System (Syngen Biotech, Wroclaw, Poland).

#### 3.2.5. Interaction with Human Serum Albumin

Human serum albumin (HSA) was dissolved in 50 mM phosphate buffer saline (PBS) (pH = 7.4), C (HSA) ≈ 5 × 10^− 5^ M. The solution of the HSA was titrated at different molar ratios (0.5, 1, 2, 3, 4, 5, 6, 7, 8, 9 and 10) by **IrPCp**, **IrPNr**, **IrPSf**, **IrPLm** and phosphine ligands (dissolved in DMSO) and incubated for 1 h with any portion from each of the investigated compounds. The final volume of DMSO was 2% in each sample. Afterward, substances were incubated with HSA solution for 1h at room temperature. The excitation wavelength was equal to 295 nm.

#### 3.2.6. Interaction with Transferring

Apo-transferrin (apo-Tf) solution was prepared by dissolving the solid protein in 50 mM of phosphate-buffered saline (PBS) (pH = 7.4), C (apo-Tf) ≈ 3,6 × 10^− 6^ M. The solution of the Tf was titrated in different molar ratios (0.5, 1, 2, 3, 4, 5, 6, 7, 8, 9, and 10) by **IrPCp**, **IrPNr**, **IrPSf**, **IrPLm** with corresponding phosphines (dissolved in DMSO) and incubated for 1h with any portion from each of the investigated compounds. The final volume of DMSO was 2% in each sample. The excitation wavelength was equal to 295 nm. The spectra of circular dichroism were recorded using a spectropolarimeter JASCO J-715 (CD and MCD).

#### 3.2.7. Molecular Docking

Docking of Ir(III) complexes was performed with the program AutoDock4 (4.2.6 Release) [67] using Lamarckian Genetic Algorithm. Preparation of ligands and receptors was conducted with the program AutoDockTools [67] using the default settings unless stated otherwise. Each docking simulation consisted of 100 independent docking runs with the maximum number of energy evaluations set to 25 × 10^6^. The original AutoDock4 parameter file was modified to incorporate iridium parameters obtained from the AutoDock website [68]. The details for particular receptors are outlined in further detail. The binding poses that resulted were clustered with a 2 Å RMSD threshold.

DNA docking was conducted using DNA hexamer d(CGATCG)_2_ (PDB code 1Z3F [69]) as the receptor. After removing the intercalating agent present in the crystal structure, two gaps facilitating possible intercalation remained in the structure. The search space described by a three-dimensional grid was set to cover the entire DNA molecule. In particular, the center of the grid was aligned with the geometric center of the receptor. The grid spacing was equal to 0.375 Å. The grid dimensions corresponded to 26.25, 26.25, and 30 Å along the x, y, and z axes, respectively.

A high-resolution crystal structure of human serum albumin (PDB code 1N5U [70]) was employed as the receptor in HSA docking. A cubic grid of 26.25 Å size and 0.375 Å spacing was centered at the side chain nitrogen atoms of Trp214 residue.

The possible binding pockets within apo-Tf structure were determined using the ligand-binding site predictions provided by DeepSite [71], ConCavity [72], and PrankWeb [73,74] tools with default settings. A crystal structure of human serum apo-transferrin (PDB code 2HAV [75]) was used as the receptor for both binding pocket prediction and further docking simulation. The number of ligand binding sites predicted with DeepSite, ConCavity, and PrankWeb was equal to 2, 3, and 6, respectively. When considering the mutual arrangement of these binding pockets, four receptor sites were selected, essentially covering all the predictions obtained with the tools applied herein. Separate docking simulations were conducted for each of the selected binding pockets. The respective four cubic grids with 26.25 Å edge length were centered at the following sets of coordinates: x_1_ = −42.818, y_1_ = −7.435, z_1_ = −21.026, x_2_ = −36.325, y_2_ = −1.776, z_2_ = 3.335, x_3_ = −23.178, y_3_ = −4.200, z_3_ = 12.921, x_4_ = −55.864, y_4_ = 3.726, and z_4_ = −18.111 (in Å units with respect to the original coordinates of 2HAV crystal structure).

## 4. Conclusions

The mode of binding of four Ir(III) complexes (Ir(η^5^-Cp*)Cl_2_Ph_2_PCH_2_Cp; **IrPCp**, Ir(η^5^-Cp*)Cl_2_Ph_2_PCH_2_Sf; **IrPSf**, Ir(η^5^-Cp*)Cl_2_Ph_2_PCH_2_Lm; **IrPLm**, Ir(η^5^-Cp*)Cl_2_Ph_2_PCH_2_Nr; **IrPNr**)) with CTDNA and serum proteins human albumin and apo-transferrin was investigated using various techniques (fluorescence spectroscopy, circular dichroism, and gel electrophoresis) and molecular docking studies.

In our investigation, we proved that: (i) Interaction of Ir(III) complexes with DNA is possible by noncovalent modes without a double-strand cleavage; (ii) DNA damage is possible via a ROS-dependent mechanism involving hydroxyl radicals, singlet oxygen, and superoxide anion; (iii) compounds interact with macromolecules such as HSA and apo-Tf; additionally, the molecular docking study indicated that all the compounds considered herein were found to (iv) bind to the tryptophan residues of HSA within site I, and/or (v) to dock within all the four predicted binding sites of apo-Tf, which include nearby tyrosine or tryptophan residues.

This experimental evidence indicates a mechanism of action different from DNA targeting typical of Pt(II) drugs. Its noticeable activity concerns the interaction with DNA with a predominance of groove binding potentially related to negligible genotoxicity, and a high level of ROS generation.

## Figures and Tables

**Table 1:** Determined Ksv values for the studied phosphines and Ir(III) complexes.

Ksv [M−1]			
	Intercalation	Minor Groove Binding	Major Groove Binding
Ligands			
PCp	7.62 × 101	7.43 × 103	0.03 × 101
PNr	5.27 × 102	1.57 × 103	0.25 × 101
PLm	2.59 × 102	1.91 × 102	0.14 × 101
PSf	2.05 × 102	7.39 × 103	0.13 × 101
Complexes			
IrPCp	1.42 × 103	1.23 × 104	3.25 × 101
IrPNr	1.54 × 103	8.25 × 103	0.46 × 101
IrPLm	1.48 × 103	6.25 × 103	2.13 × 101
IrPSf	1.51 × 103	1.36 × 104	0.41 × 101

**Table 2:** The percentage of binding poses representing the particular binding mode in DNA-phosphino iridium(III) complexes for all the docking results (“All clusters”) or the largest clusters (“Clusters with at least six members”).

	IrPCp		IrPNr		IrPLm		IrPSf	
	All Clusters	Clusters with at Least 6 Members	All Clusters	Clusters with at Least 6 Members	All Clusters	Clusters with at Least 6 Members	All Clusters	Clusters with at Least 6 Members
Minor groove binding	47%	59%	48%	42%	60%	89%	47%	81%
Intercalat ion and major groove binding	41%	41%	44%	58%	34%	11%	36%	19%
Intercalat ion and minor groove binding	12%	-	8%	-	6%	-	17%	-
Number of binding poses	100	61	100	53	100	53	100	37

**Table 3:** Determined Ksv values for the studied phosphines and Ir(III) complexes.

Ksv [M^−1^]			
PCp	0.21 × 10^1^	IrPCp	2.08 × 10^6^
PSf	0.11 × 10^1^	IrPSf	5.67 × 10^6^
PLm	0.53 × 10^1^	IrPLm	5.06 × 10^6^
PNr	0.19 × 10^1^	IrPNr	6.98 × 10^5^

**Table 4:** Determined Ksv values for the interactions of studied phosphine and Ir(III) complexes.

Ksv [M^−1^]			
PCp	0.45 × 10^1^	IrPCp	2.18 × 10^4^
PSf	1.10 × 10^4^	IrPSf	1.32 × 10^4^
PLm	1.52 × 10^2^	IrPLm	1.99 × 10^4^
PNr	4.56 × 10^1^	IrPNr	2.02 × 10^4^

## Data Availability

Data is contained within the article.

## Supplementary Materials

The following are available online at https://www.mdpi.com/article/10.3390/ph14070685/s1, Figure S1. Fluorescence quenching of EB–CT DNA (C = 5 × 10^−5^ M) by IrPCp, IrPNr, IrPLm and IrPSf (molar ratios 0.5, 1, 1.5, 2, 3, 4, 5, 6, 7, 8, 9 and 10) in 50 mM pH 7.4 phosphate buffer (axis: y—fluorescence intensity; x—wavelength), Figure S2. Fluorescence quenching of DAPI-CT DNA (C = 5 × 10^−5^ M) by IrPCp, IrPNr, IrPLm and IrPSf (molar ratios 0.5, 1, 1.5, 2, 3, 4, 5, 6, 7, 8, 9, 10) in 50 mM pH 7.4 phosphate buffer (axis: y—fluorescence intensity; x—wavelength), Figure S3. Fluorescence quenching of DAPI-CT DNA (C = 5 × 10^−5^ M) by PCp, PNr, PLm and PSf (molar ratios 0.5, 1, 1.5, 2, 3, 4, 5, 6, 7, 8, 9, 10) in 50 mM pH 7.4 phosphate buffer (axis: y—fluorescence intensity; x—wavelength), Figure S4. Fluorescence quenching of DAPI-CT DNA (C = 5 × 10^−5^ M) by IrPCp, IrPNr, IrPLm and IrPSf (molar ratios 0.5, 1, 1.5, 2, 3, 4, 5, 6, 7, 8, 9, 10) in 50 mM pH 7.4 phosphate buffer (axis: y—fluorescence intensity; x—wavelength), Figure S5. Fluorescence quenching of HSA (C = 5 × 10^−5^ M) by IrPCp, IrPNr, IrPLm and IrPSf (molar ratios 0.5, 1, 1.5, 2) in 50 mM pH 7.4 phosphate buffer (axis: y—fluorescence intensity; x—wavelength), Figure S6. Fluorescence quenching of apo-Tf (C = 3.6 × 10^−6^ M) by IrPCp, IrPNr, IrPLm and IrPSf (molar ratios 0.5, 1, 1.5, 2, 3, 4, 5, 6, 7, 8, 9, 10) in 50 mM pH 7.4 phosphate buffer (axis: y—fluorescence intensity; x—wavelength), Figure S7. Fluorescence quenching of apo-Tf (C = 3.6 × 10^−6^ M) by PCp, PNr, PLm and PSf (molar ratios 0.5, 1, 1.5, 2, 3, 4, 5, 6, 7, 8, 9, 10) in 50 mM pH 7.4 phosphate buffer (axis: y—fluorescence intensity; x—wavelength). Figure S8. Agarose gel electrophoresis of pBR322 plasmid cleavage by H_2_O_2_ in different concentrations.
