# Supplementary material for: Interaction between DNA, Albumin and Apo-Transferrin and Iridium(III) Complexes with Phosphines Derived from Fluoroquinolones as a Potent Anticancer Drug
Source: Pharmaceuticals (Basel). 2021 Jul 16;14(7):685. doi: 10.3390/ph14070685 (PMC8308524; doi:10.3390/ph14070685)
Supplement: Supplementary file 1 [file pharmaceuticals-14-00685-s001.zip › pharmaceuticals-1289686-supplementary.pdf]

Multi experimental and molecular docking studies on the DNA, albumin and apo-transferrin interaction with phosphino iridium(III) complexes as a potent anticancer drug.

Sandra Kozieł[a*], DariaWojtala,[a] Monika K. Lesiów,[a]Edyta Dyguda-Kazimierowicz,[b]Dariusz C. Bieńko,[b] Urszula K. Komarnicka[a*]

[a] Faculty of Chemistry, University of Wroclaw, Joliot-Curie 14, 50-383 Wroclaw, Poland

[b] Faculty of Chemistry, Wroclaw University of Science and Technology, WybrzeżeWyspiańskiego 27, 50-370 Wroclaw, Poland

* Correspondence: authors e-mail address: urszula.komarnicka@chem.uni.wroc.pl, sandra.koziel@chem.uni.wroc.pl

ABSTRACT: Group of cytotoxic half-sandwich iridium(III) complexes with aminomethyl(diphenyl)phosphine derived from fluoroquinolone antibiotics exhibits ability to (*i*) accumulate in nucleus, (*ii*) induce apoptosis, (*iii*) activate caspase-3/7 activity, (*iv*) induce the changes in cell cycle leading to G2/M phase arrest and (*v*) radicals generation. Herein, to elucidate the cytotoxic effects, we investigated the interaction of these complexes with DNA and serum proteins by gel electrophoresis, fluorescence spectroscopy, circular dichroism, and molecular docking studies. DNA binding experiments established that the complexes interact with DNA by moderate intercalation and predominance of minor groove binding without any capability to cause a double-strand cleavage. Molecular docking study confirmed two binding modes: minor groove binding and threading intercalation with fluoroquinolone part of the molecule involved in pi stacking interactions and the Ir(III)-containing region positioned within the major or minor groove. Fluorescence spectroscopic data (HSA and apo-Tf titration), together with molecular docking provided evidence that Ir(III) complexes are able to bind to the proteins in order to be transferred. All the compounds considered herein were found to bind to the tryptophan residues of HSA within site I (subdomain II A). Furthermore, Ir(III) complexes were found to dock within the apo-Tf binding site, which includes nearby tyrosine residues.

Keywords: arene iridium(III) complexes; fluoroquinolones; DNA-binding studies; DNA cleavage; proteins-binding studies; drug delivery;reactive oxygen species; anticancer activity

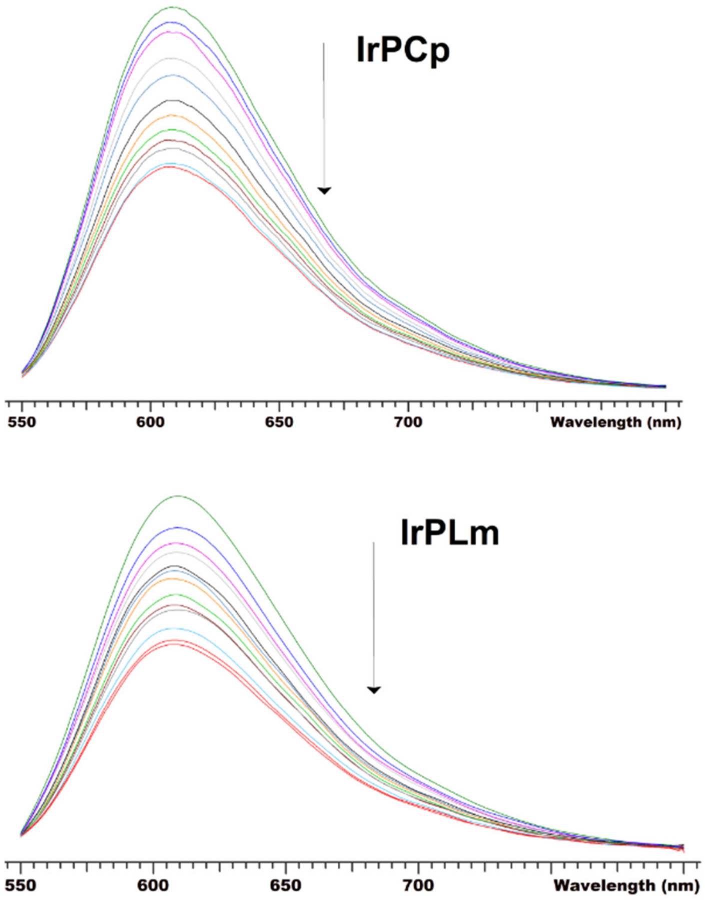

Figure S1. Fluorescence quenching of EB–CT DNA (C = 5×10^{-5} M) by **IrPCp**, **IrPNr**, **IrPLm** and **IrPSf** (molar ratios 0.5, 1, 1.5, 2, 3, 4, 5, 6, 7, 8, 9 and 10) in 50 mM pH 7.4 phosphate buffer (axis: y – fluorescence intensity; x – wavelength).

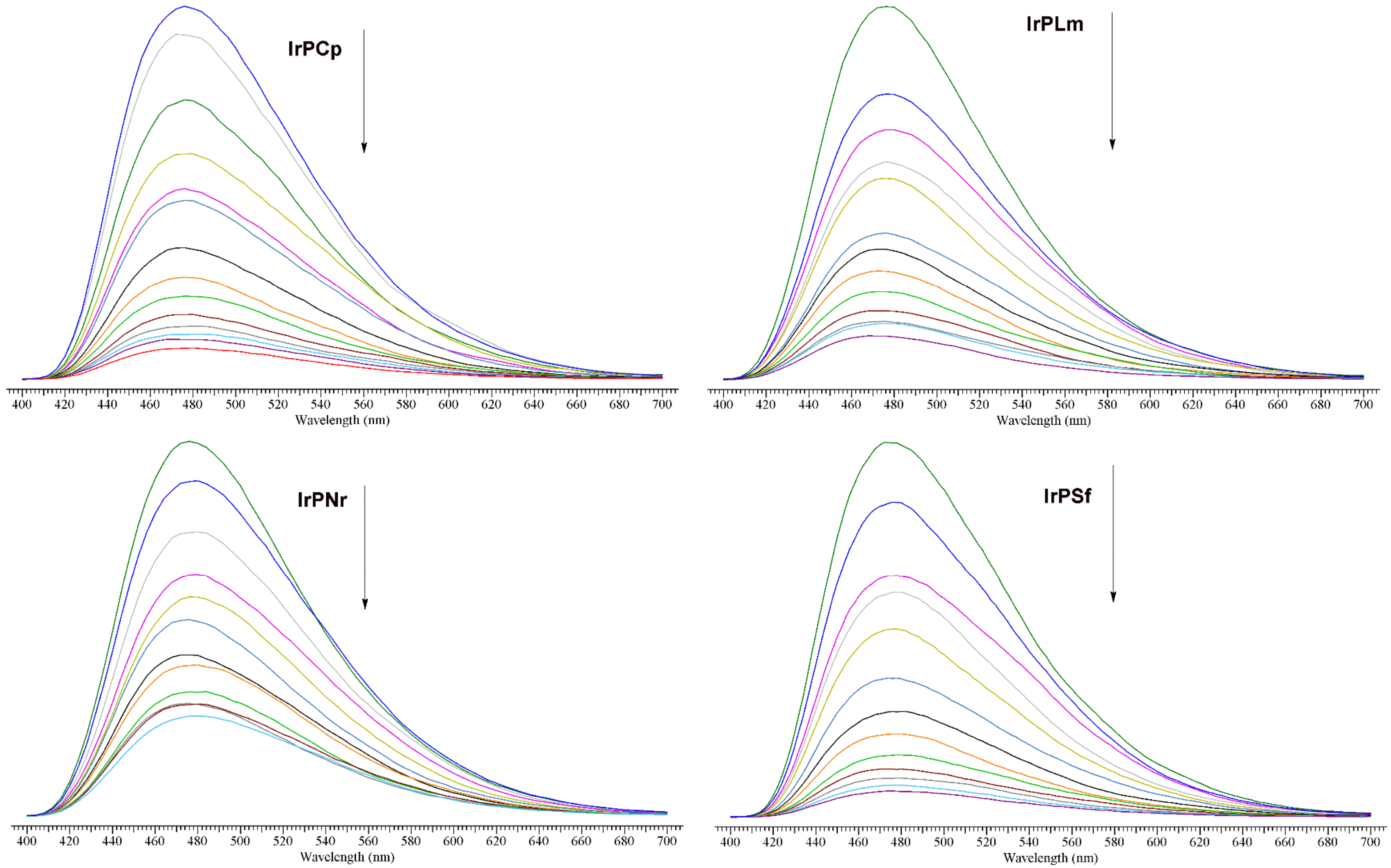

Figure S2. Fluorescence quenching of DAPI-CT DNA (C = 5 × 10^{-5} M) by **IrPCp**, **IrPNr**, **IrPLm** and **IrPSf** (molar ratios 0.5, 1, 1.5, 2, 3, 4, 5, 6, 7, 8, 9, 10) in 50 mM pH 7.4 phosphate buffer (axis: y – fluorescence intensity; x – wavelength).

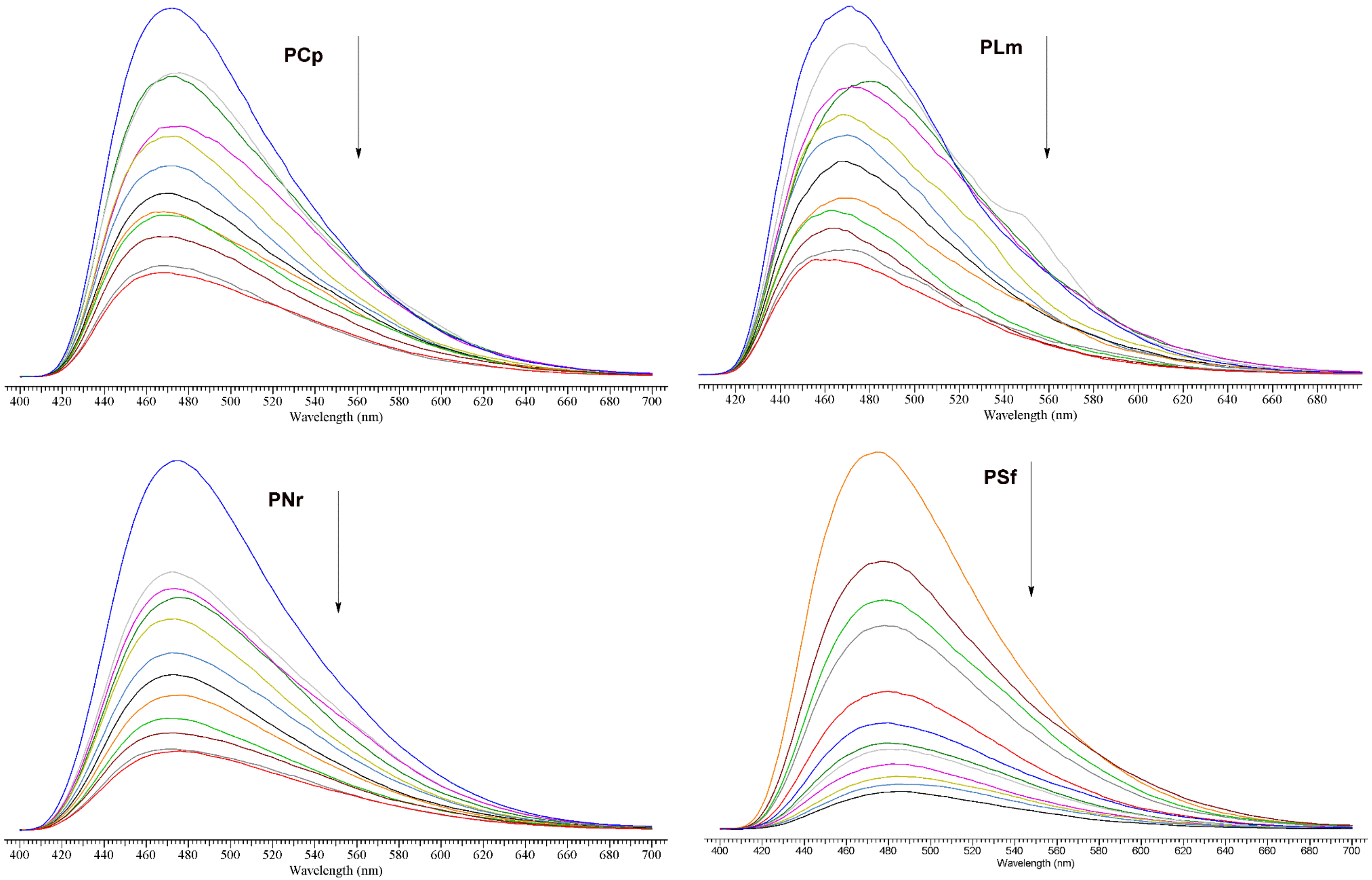

Figure S3. Fluorescence quenching of DAPI-CT DNA (C = 5 × 10^{-5} M) by **PCp**, **PNr**, **PLm** and **PSf** (molar ratios 0.5, 1, 1.5, 2, 3, 4, 5, 6, 7, 8, 9, 10) in 50 mM pH 7.4 phosphate buffer (axis: y – fluorescence intensity; x – wavelength).

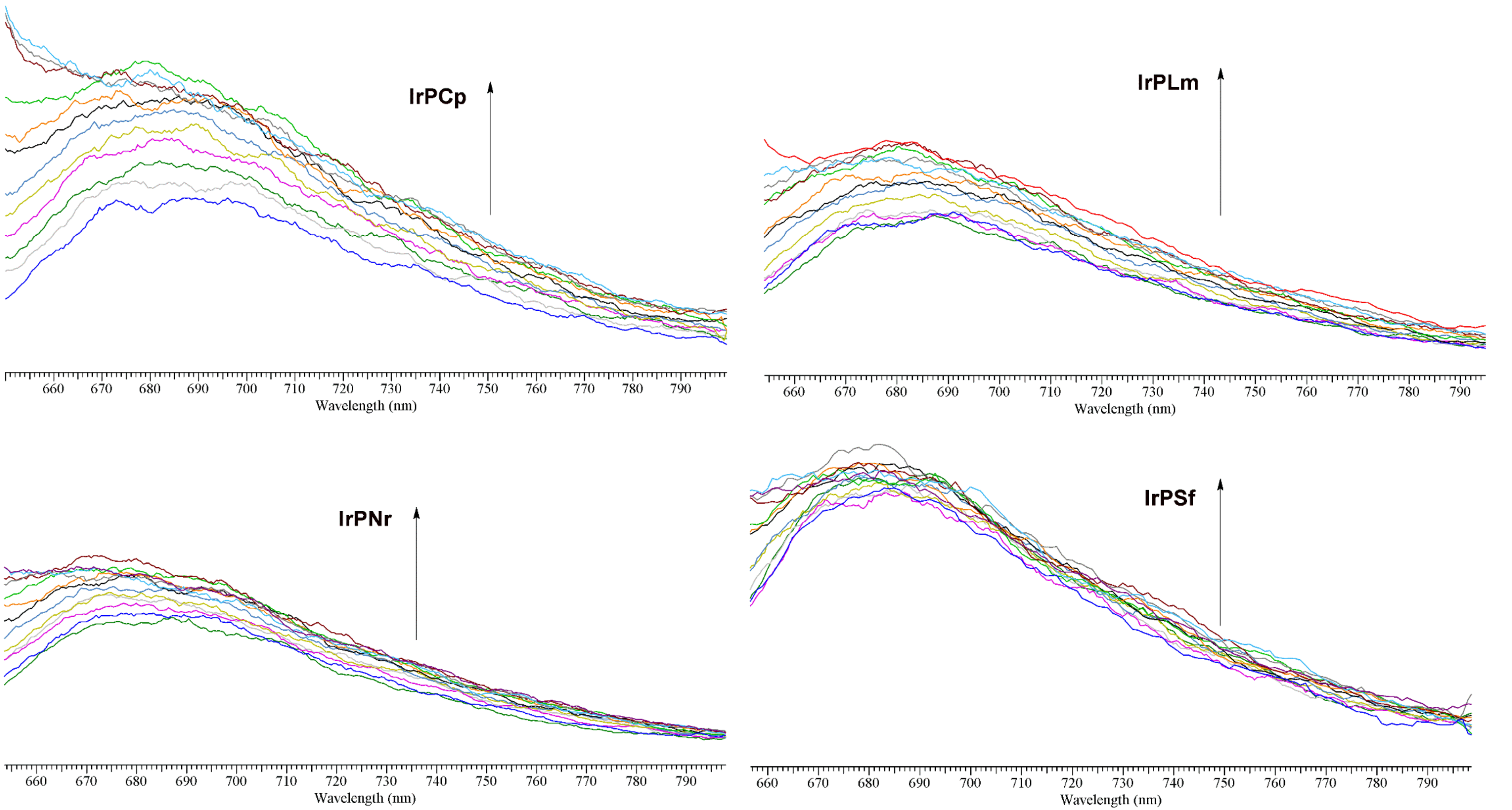

Figure S4. Fluorescence quenching of DAPI-CT DNA (C = 5 × 10^{-5} M) by **IrPCp**, **IrPNr**, **IrPLm** and **IrPSf** (molar ratios 0.5, 1, 1.5, 2, 3, 4, 5, 6, 7, 8, 9, 10) in 50 mM pH 7.4 phosphate buffer (axis: y – fluorescence intensity; x – wavelength).

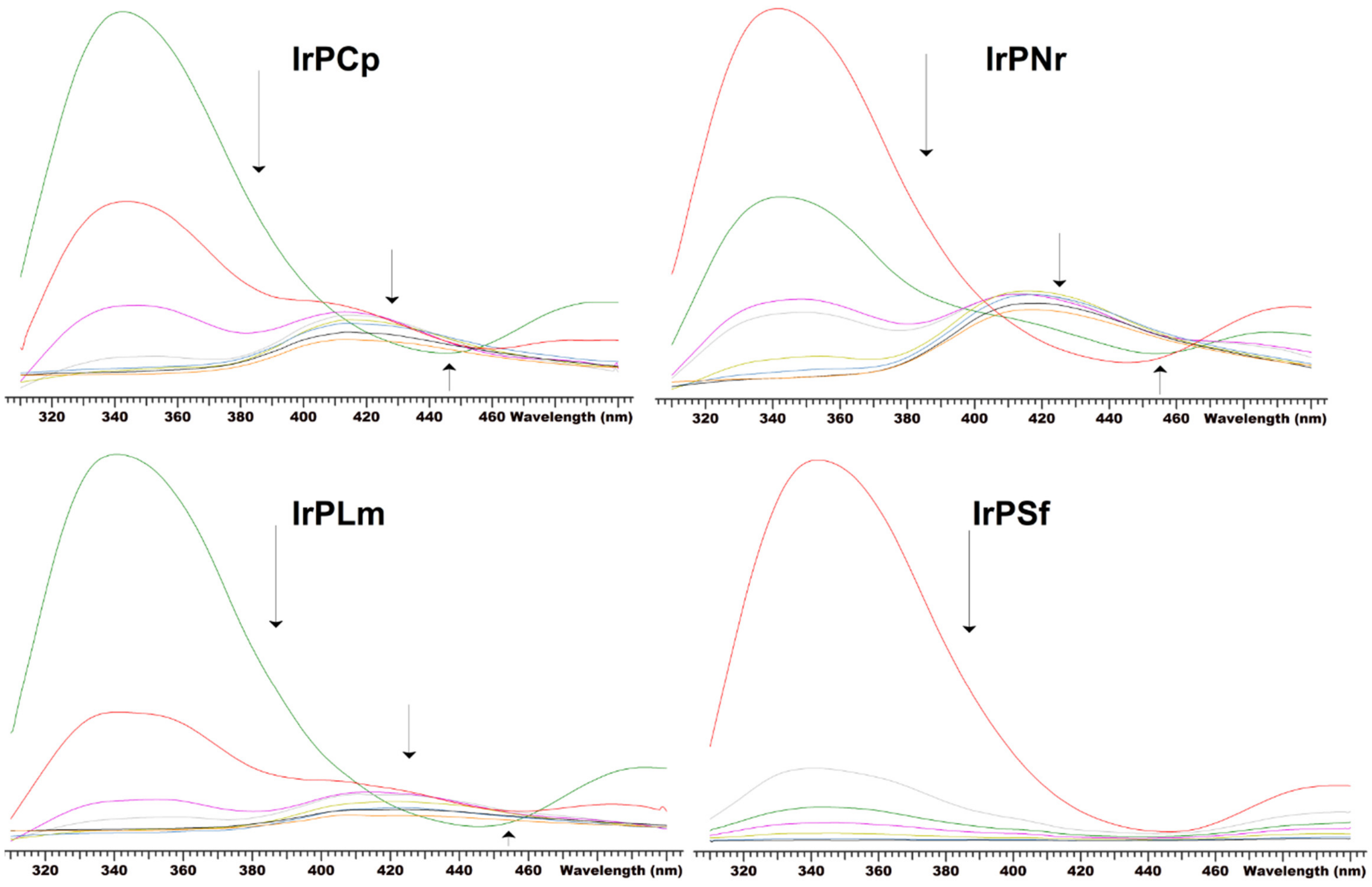

Figure S5. Fluorescence quenching of HSA (C = 5 × 10^{-5}M) by **IrPCp**, **IrPNr**, **IrPLm** and **IrPSf** (molar ratios 0.5, 1, 1.5, 2) in 50 mM pH 7.4 phosphate buffer (axis: y – fluorescence intensity; x – wavelength).

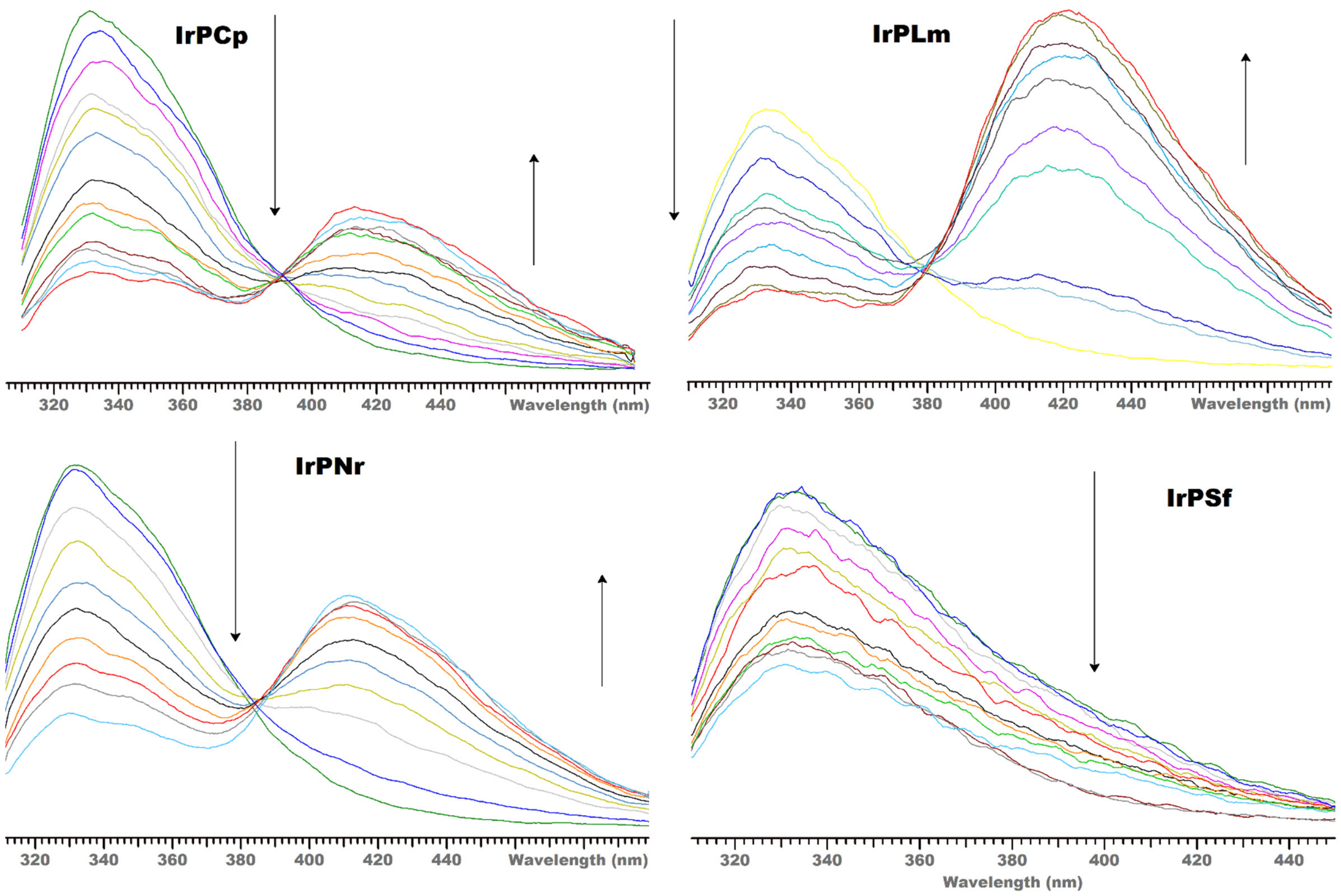

Figure S6. Fluorescence quenching of apo-Tf (C = 3.6 × 10^{-6} M) by **IrPCp**, **IrPNr**, **IrPLm** and **IrPSf** (molar ratios 0.5, 1, 1.5, 2, 3, 4, 5, 6, 7, 8, 9, 10) in 50 mM pH 7.4 phosphate buffer (axis: y – fluorescence intensity; x – wavelength).

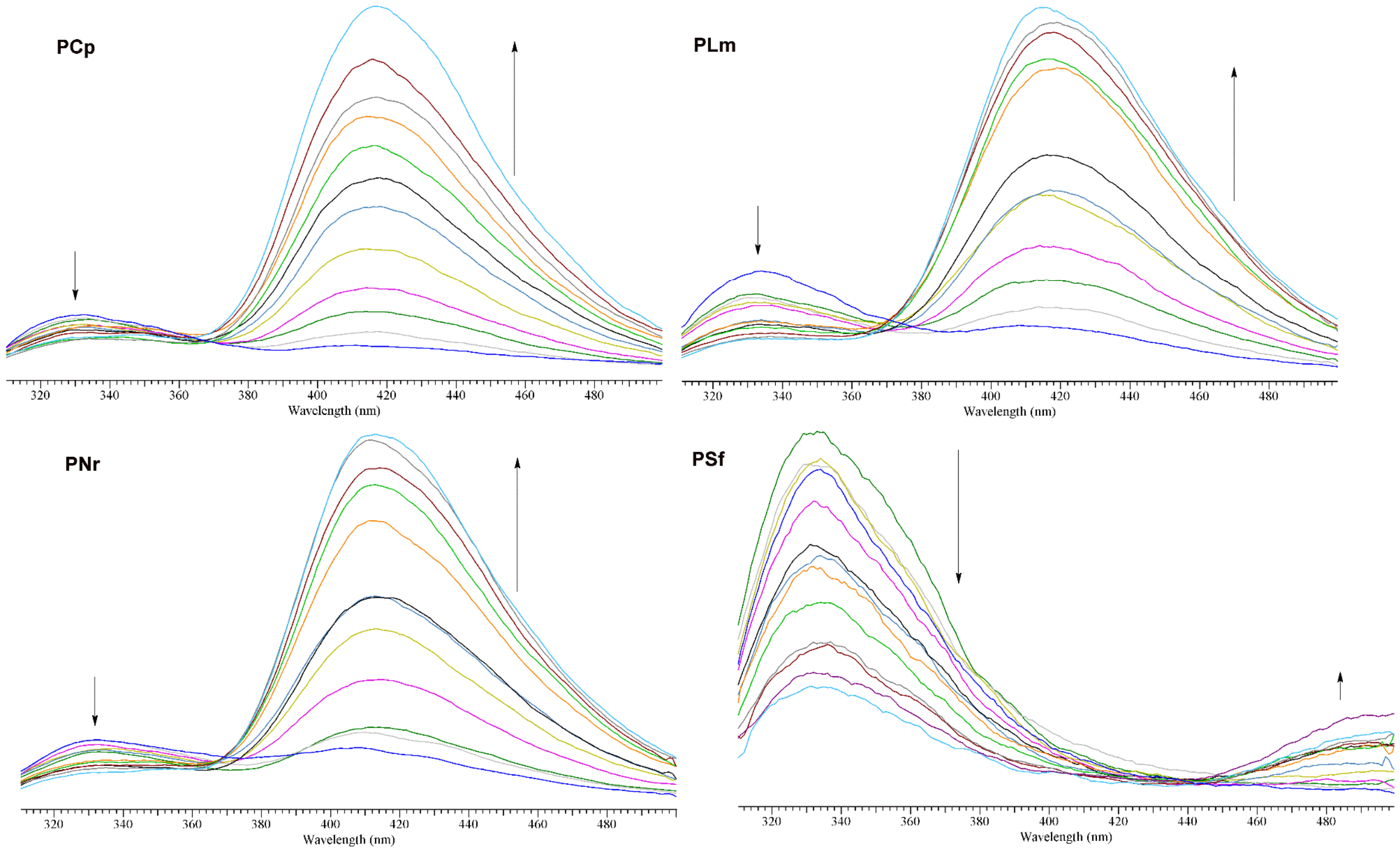

Figure S7. Fluorescence quenching of apo-Tf (C = 3.6 × 10^{-6} M) by **PCp**, **PNr**, **PLm** and **PSf** (molar ratios 0.5, 1, 1.5, 2, 3, 4, 5, 6, 7, 8, 9, 10) in 50 mM pH 7.4 phosphate buffer (axis: y – fluorescence intensity; x – wavelength).

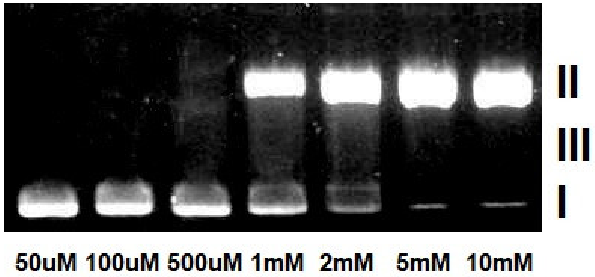

Figure S8. Agarose gel electrophoresis of pBR322 plasmid cleavage by H_2O_2 in different concentrations.
